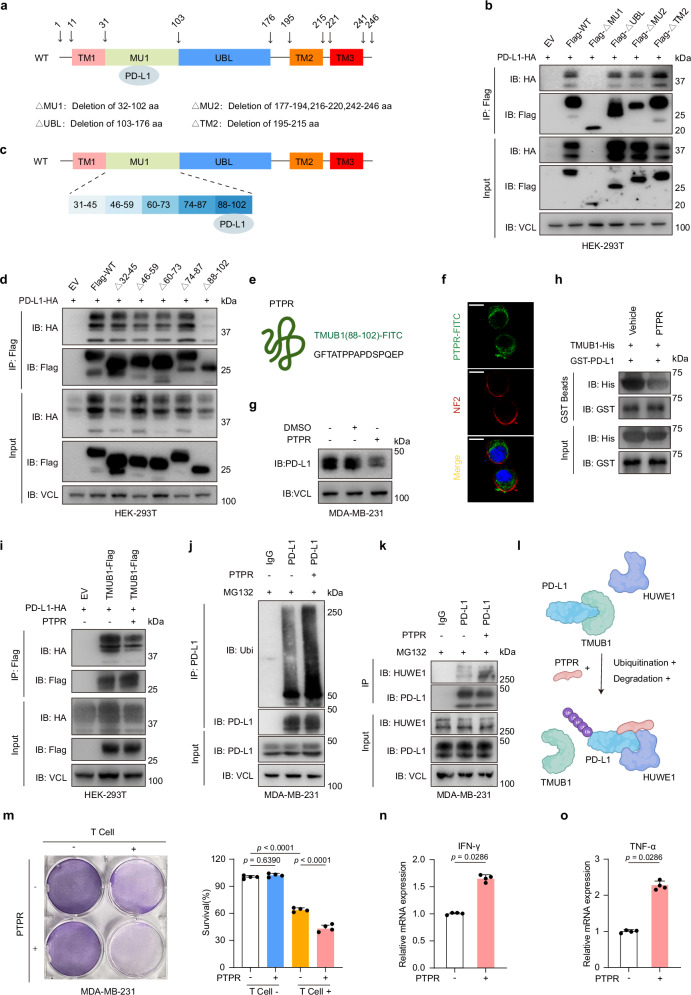# Author Correction: Promoting anti-tumor immunity by targeting TMUB1 to modulate PD-L1 polyubiquitination and glycosylation

**DOI:** 10.1038/s41467-024-55049-5

**Published:** 2024-12-10

**Authors:** Chengyu Shi, Ying Wang, Minjie Wu, Yu Chen, Fangzhou Liu, Zheyuan Shen, Yiran Wang, Shaofang Xie, Yingying Shen, Lingjie Sang, Zhen Zhang, Zerui Gao, Luojia Yang, Lei Qu, Zuozhen Yang, Xinyu He, Yu Guo, Chenghao Pan, Jinxin Che, Huaiqiang Ju, Jian Liu, Zhijian Cai, Qingfeng Yan, Luyang Yu, Liangjing Wang, Xiaowu Dong, Pinglong Xu, Jianzhong Shao, Yang Liu, Xu Li, Wenqi Wang, Ruhong Zhou, Tianhua Zhou, Aifu Lin

**Affiliations:** 1 MOE Laboratory of Biosystem Homeostasis and Protection, College of Life Sciences, Zhejiang University, Hangzhou, Zhejiang 310058 China; 2 Cancer Center, Zhejiang University, Hangzhou, Zhejiang 310058 China; 3 Key Laboratory for Cell and Gene Engineering of Zhejiang Province, Hangzhou, Zhejiang 310058 China; 4 Innovation Institute for Artificial Intelligence in Medicine, Zhejiang University, Hangzhou, Zhejiang 310016 China; 5 Hangzhou Institute of Innovative Medicine, Institute of Drug Discovery and Design, College of Pharmaceutical Sciences, Zhejiang University, Hangzhou, Zhejiang 310058 China; 6 Key Laboratory of Structural Biology of Zhejiang Province, Westlake Laboratory of Life Sciences and Biomedicine, Westlake University, Hangzhou, Zhejiang 310024 China; 7 Institute of Immunology, Zhejiang University School of Medicine, Hangzhou, Zhejiang 310009 China; 8 Sun Yat-sen University Cancer Center, State Key Laboratory of Oncology in South China, Collaborative Innovation Center for Cancer Medicine, Guangzhou, Guangdong 510060 China; 9 Zhejiang University-University of Edinburgh Institute (ZJU-UoE Institute), Zhejiang University School of Medicine, International Campus, Zhejiang University, Haining, Zhejiang 314400 China; 10 Department of Gastroenterology, the Second Affiliated Hospital, School of Medicine and Institute of Gastroenterology, Zhejiang University, Hangzhou, Zhejiang China; 11 MOE Laboratory of Biosystems Homeostasis & Protection and Zhejiang Provincial Key Laboratory for Cancer Molecular Cell Biology, Life Sciences Institute, Zhejiang University, Hangzhou, Zhejiang 310058 China; 12 Department of Developmental and Cell Biology, University of California, Irvine; Irvine, CA 92697 USA; 13 Shanghai Institute for Advanced Study, Zhejiang University, 201203 Shanghai, China; 14 Department of Chemistry, Colombia University, New York City, NY 10027 USA; 15 Institute of Quantitative Biology, Zhejiang University, Hangzhou, Zhejiang 310058 China; 16 Department of Cell Biology and Program in Molecular Cell Biology, Zhejiang University School of Medicine, Hangzhou, Zhejiang 310058 China; 17 Department of Gastroenterology, the Second Affiliated Hospital, School of Medicine and Institute of Gastroenterology, Zhejiang University, Hangzhou, Zhejiang 310009 China; 18 Breast Center of the First Affiliated Hospital, School of Medicine, Zhejiang University, Hangzhou, Zhejiang 310003 China; 19 International School of Medicine, International Institutes of Medicine, The 4th Affiliated Hospital of Zhejiang University School of Medicine, Yiwu, Zhejiang 322000 China; 20 ZJU-QILU Joint Research Institute, Hangzhou, Zhejiang 310058 China

**Keywords:** Tumour immunology, Post-translational modifications, Immunotherapy, Protein transport

Correction to: *Nature Communications* 10.1038/s41467-022-34346-x, published online 14 November 2022

“The original version of this Article contained an error in Fig. 6k, in which a duplicate western blot image for PD-L1 from Fig. 3j was re-used inadvertently. This has been corrected in both the PDF and HTML versions of the Article, as well as in the Source Data file. Both the incorrect and correct versions of Fig. 6k are shown below.”

Incorrect figure


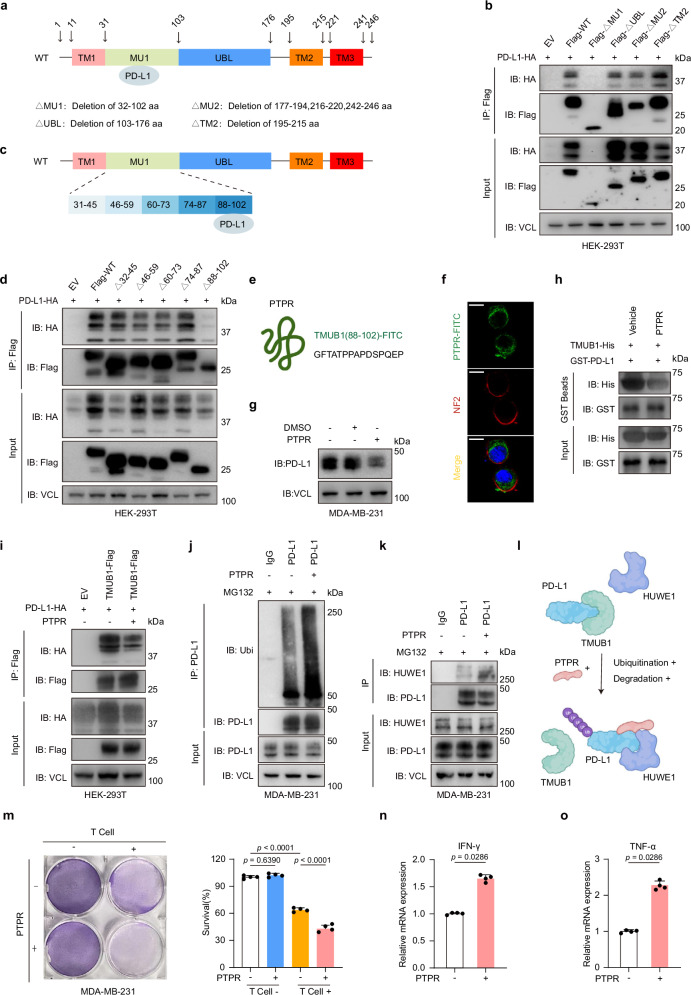


Corrected figure