# Supplementary material for: Promoting anti-tumor immunity by targeting TMUB1 to modulate PD-L1 polyubiquitination and glycosylation
Source: Nat Commun. 2022 Nov 14;13:6951. doi: 10.1038/s41467-022-34346-x (PMC9663433; doi:10.1038/s41467-022-34346-x)
Supplement: Supplementary file 1 — Supplementary Information [file 41467_2022_34346_MOESM1_ESM.pdf]

Promoting anti-tumor immunity by targeting TMUB1, a modulator of PD-L1 polyubiquitination and glycosylation

(Supplementary Information)

Chengyu Shi[1,2,3#], Ying Wang[1,2,3#], Minjie Wu[1,2,3#], Yu Chen[1,2,3], Fangzhou Liu[1,2,3], Zheyuan Shen[4,5], Yiran Wang[1], Shaofang Xie[6], Yingying Shen[7], Lingjie Sang[1], Zhen Zhang[1], Zerui Gao[1], Luojia Yang[1], Lei Qu[1], Zuozhen Yang[1], Xinyu He[1], Yu Guo[5], Chenghao Pan[4,5], Jinxin Che[5], Huaiqiang Ju[8], Jian Liu[9], Zhijian Cai[7], Qingfeng Yan[1], Luyang Yu[1], Liangjing Wang[10], Xiaowu Dong[4,5], Pinglong Xu[11], Jianzhong Shao[1], Yang Liu[7], Xu Li[6*], Wenqi Wang[12*], Ruhong Zhou[1,2,13,14,15*], Tianhua Zhou[2,16,17*] and Aifu Lin[1,2,3,18,19,20*]

[#]These authors have contributed equally.

*To whom correspondence should be addressed: linaifu@zju.edu.cn, tzhou@zju.edu.cn, wenqiw6@uci.edu, lixu@westlake.edu.cn and rhzhou@zju.edu.cn.

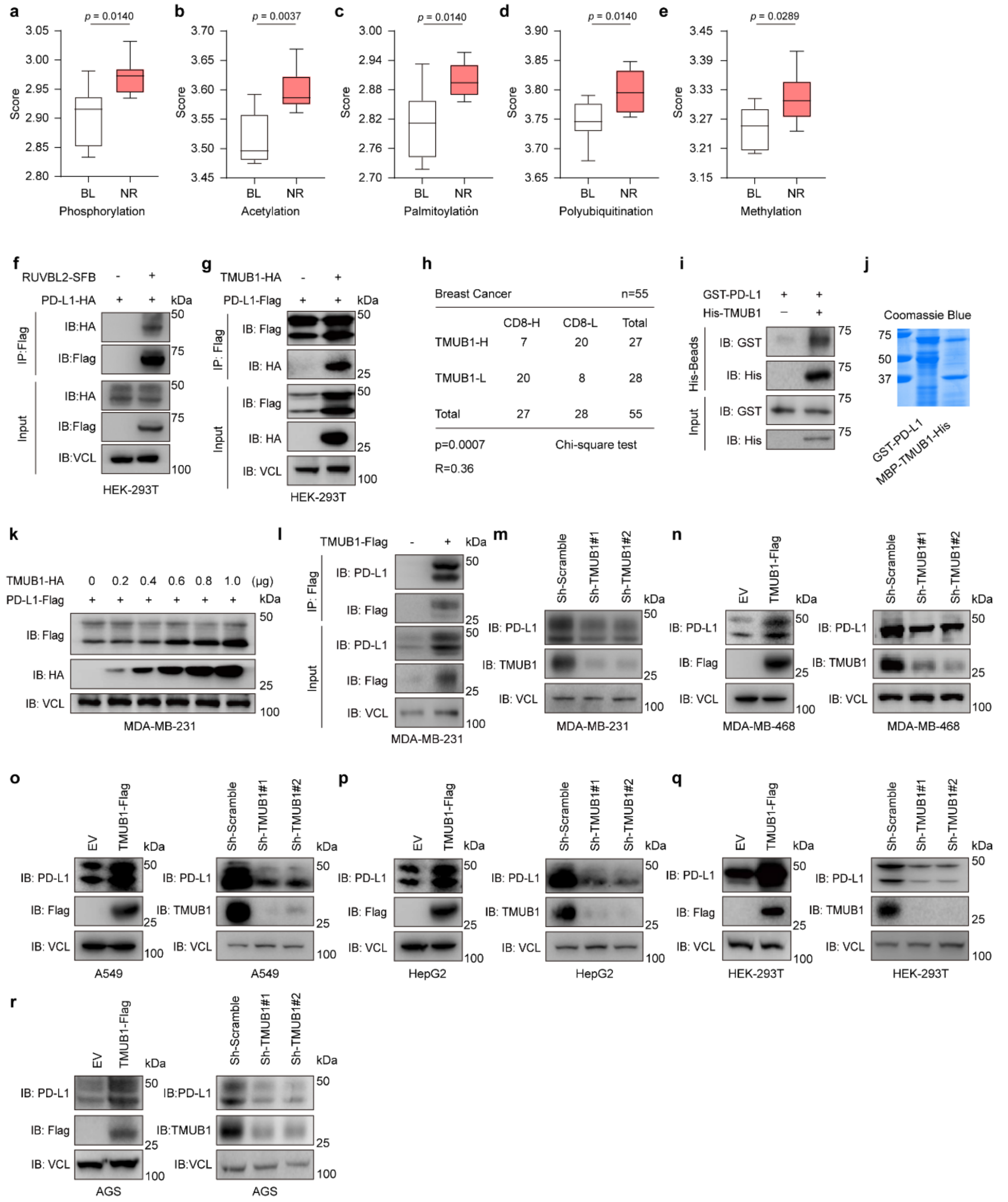

Supplementary Fig. 1, related to Fig. 1 TMUB1 was identified as a positive regulator of PD-L1.

(a-e) GO biological process analysis of phosphorylation **(a)**, acetylation **(b),** palmitoylation **(c)**, polyubiquitination **(d)** and methylation **(e)** pathway in baseline (BL) and non-responder (NR) of anti-PD-L1 treatment in 4T1 breast cancer mouse model from TISMO (http://tismo.cistrome.org/). The differences between groups are

statistically evaluated by the Two-sided Wilcox test, n = 15. **(f)** Co-IP analysis of the interaction between PD-L1-HA and RUVBL2-SFB (Protein S-Flag-SBP) within HEK-293T cell line. **(g)** Co-IP analysis for the interaction between PD-L1-Flag and TMUB1-HA in HEK-293T cells. **(h)** The expressions of TMUB1 and infiltration of $CD8^+$ T cells in 55 primary human breast cancer specimens (SYSUCC cohorts, n=55) were detected using the IHC assay. The correlation between the two was analyzed using a Two-sided chi-square test. **(i)** Recombinant PD-L1-GST and TMUB1-His were purified for use in the His pull-down assay. The interaction between TMUB1 and PD-L1 was detected using the immunoblot assay. **(j)** The Coomassie staining gel of purified GST-PD-L1 and MBP-TMUB1-His. **(k)** Immunoblots of PD-L1-Flag in the MDA-MB-231 cells transfected with a gradient (increasing concentrations 0 to 1.0 ug) of TMUB1-HA. **(l)** Co-IP analysis of the interaction between TMUB1-Flag and endogenous PD-L1 in MDA-MB-231 cells. **(m)** Immunoblots of PD-L1 and TMUB1 in control or TMUB1-knockdown in the MDA-MB-231 cells. **(n)** Immunoblots of PD-L1 and TMUB1 in TMUB1-overexpression (left), TMUB1-knockdown (right) or control MDA-MB-468 cells. **(o)** Immunoblots of PD-L1 and TMUB1 in TMUB1-overexpression (left), TMUB1-knockdown (right) or control A549 cells. **(p)** Immunoblots of PD-L1 and TMUB1 in TMUB1-overexpression (left), TMUB1-knockdown (right) or control HepG2 cells. **(q)** Immunoblots of PD-L1 and TMUB1 in TMUB1-overexpression (left), TMUB1-knockdown (right) or control HEK-293T cells. **(r)** Immunoblots of PD-L1 and TMUB1 in TMUB1-overexpression (left), TMUB1-knockdown (right) or control AGS cells.

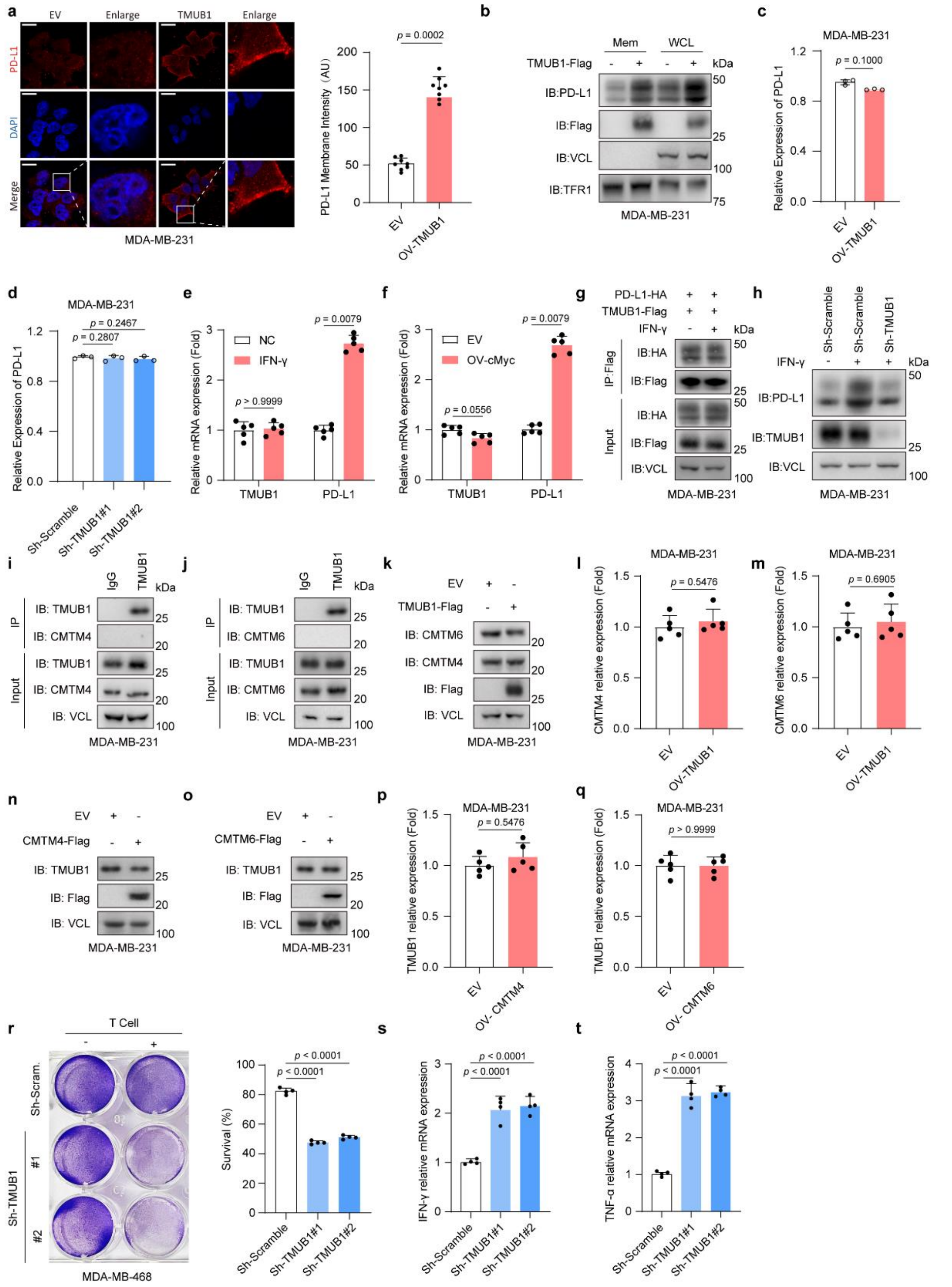

Supplementary Fig. 2, related to Fig. 1 TMUB1 was identified as a positive regulator of PD-L1.

(a) PD-L1 in the TMUB1-overexpression or control MDA-MB-231 cells detected by immunofluorescence staining. Data are presented as means ± SEM. of n = 8; Two-sided

Mann-Whitney test, Scale bar: 10 μm. **(b)** Immunoblots of PD-L1\Flag\Vinculin\TFR1 in different fractions of stable TMUB1-Flag or empty vector-Flag (EV)-overexpression MDA-MB-231 cells. TFR1 is used as the marker of the cell membrane. **(c-d)** The mRNA expression of PD-L1 in TMUB1-overexpression **(c)**, TMUB1-knockdown **(d)** or control MDA-MB-231 cells. Data presented as mean ± SEM. of n=3. Mann-Whitney test; **(e-f)** The mRNA expression of TMUB1 and PD-L1 in the MDA-MB-231 cells with stimulation of IFN-γ **(e)** or c-Myc-overexpression **(f)**. Data presented as mean ± SEM. Two-sided Mann-Whitney test, n = 5. **(g)** Co-IP analysis of the interaction between TMUB1-Flag and PD-L1 HA in MDA-MB-231 cells with stimulation of IFN-γ. **(h)** Immunoblots of PD-L1 in TMUB1-knockdown or control MDA-MB-231 cells with stimulation of IFN-γ. **(i-j)** Co-IP analysis of the interaction between endogenous TMUB1 and CMTM4 **(i)** or CMTM6 **(j)** within MDA-MB-231 cells. **(k)** Immunoblots of CMTM4 and CMTM6 in the MDA-MB-231 cells with EV or TMUB1-Flag expression. **(l-m)** The mRNA expression of CMTM4 **(l)** or CMTM6 **(m)** in the TMUB1-overexpression or control MDA-MB-231 cells. Data presented as mean ± SEM. Two-sided Mann-Whitney test, n=5. **(n-o)** Immunoblots of TMUB1 in the MDA-MB-231 cells with CMTM4-Flag **(n)** or CMTM6-Flag **(o)** overexpression. **(p-q)** The mRNA expression of TMUB1 in the MDA-MB-231 cells with overexpression of CMTM4 **(p)**, CMTM6 **(q)** or vector. Data presented as mean ± SEM. Two-sided Mann-Whitney test, n=5. **(r)** The sh-scramble or sh-TMUB1 MDA-MB-468 cells were co-cultured with activated T cells for 48 hr. The ratio of MDA-MB-468 cells to T cells: 1:5. Data presented as mean ± SEM. of n = 4. One-way ANOVA followed by Tukey

test. **(s-t)** The mRNA expressions of IFN-γ **(s)** or TNF-α **(t)** in PBMCs after co-culture with MDA-MB-468 cells. Data presented as mean ± SEM. of n = 4. One-way ANOVA followed by Tukey test.

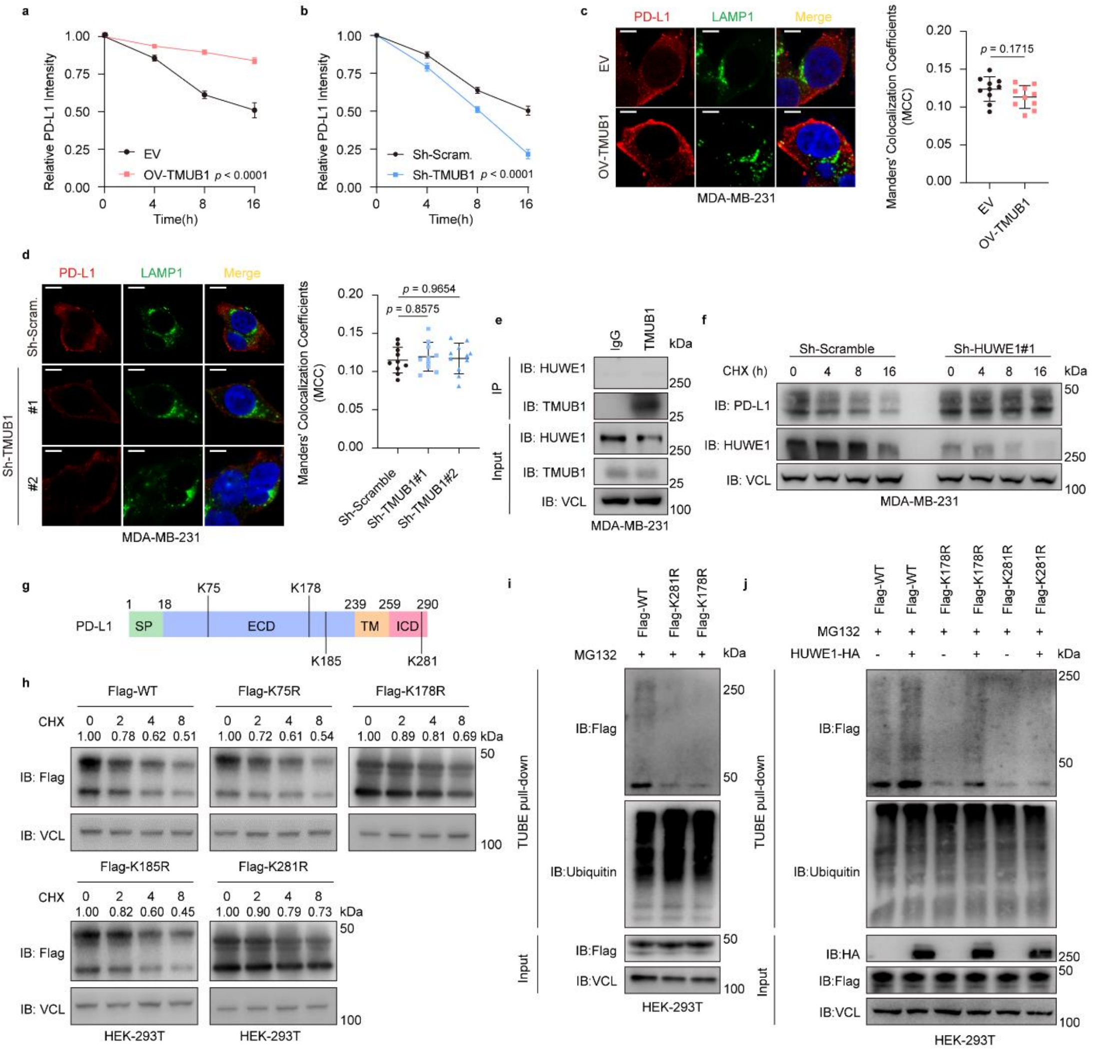

Supplementary Fig. 3, related to Fig. 2. TMUB1 stabilized PD-L1 by antagonizing its polyubiquitination mediated by the E3 ligase HUWE1

(a) Quantification of the intensity determined by the relative level of PD-L1 remaining in **Fig. 2a**. Data are presented as mean ± SEM. of n = 3. Two-way ANOVA. **(b)** Quantification of the intensity determined by the relative level of PD-L1 remaining in **Fig. 2B**. Data are presented as mean ± SEM. of n = 3. Two-way ANOVA. **(c)** PD-L1 and LAMP1 in the MDA-MB-231 cells with stable Flag-tagged empty vector (EV) or TMUB1-Flag expression detected using immunofluorescence staining. Manders'

Colocalization Coefficients (MCC) was calculated as the light intensity of LAMP1-colocalized PD-L1 divided by the light intensity of total PD-L1. Data are presented as mean ± SEM. of n = 10 biologically independent cells; One-way ANOVA followed by Tukey test. **(d)** PD-L1 and LAMP1 in control or TMUB1-knockdown MDA-MB-231 cells were detected using immunofluorescence staining. MCC was calculated as the light intensity of LAMP1-colocalized PD-L1 divided by the light intensity of total PD-L1. Data are presented as mean ± SEM. of n = 10 biologically independent cells. One-way ANOVA followed by Tukey test; Scale bar: 5 μm. **(e)** Co-IP analysis for the interaction between endogenous TMUB1 and HUWE1 in MDA-MB-231 cells. **(f)** Immunoblots of PD-L1 in sh-HUWE1 or sh-Scramble MDA-MB-231 cells following treatment with 20 μg/mL cycloheximide (CHX) for the indicated time points. **(g)** The schematic diagram for the potential ubiquitination sites on PD-L1. Green: Signal peptide, position 1–18; Blue: Extracellular domain, position 19–238; Orange: Transmembrane domain, position 239–259; Red: Cytoplasmic domain, position 260–290; Black bar: the potential ubiquitination sites. **(h)** Immunoblots of the wild-type and other mutants of PD-L1 in HEK-293T cells following treatment with 20 μg/mL of cycloheximide (CHX) for the indicated durations. **(i)** TUBE-pull-down analysis of the interaction between PD-L1-Wild Type (WT) or K281R/K178R mutant-Flag and ubiquitin in HEK-293T cells. **(j)** TUBE-pull-down analysis of the interaction between PD-L1-Wild Type (WT) or K281R/K178R mutant-Flag and ubiquitin with or without HUWE1-overexpression in HEK-293T cells.

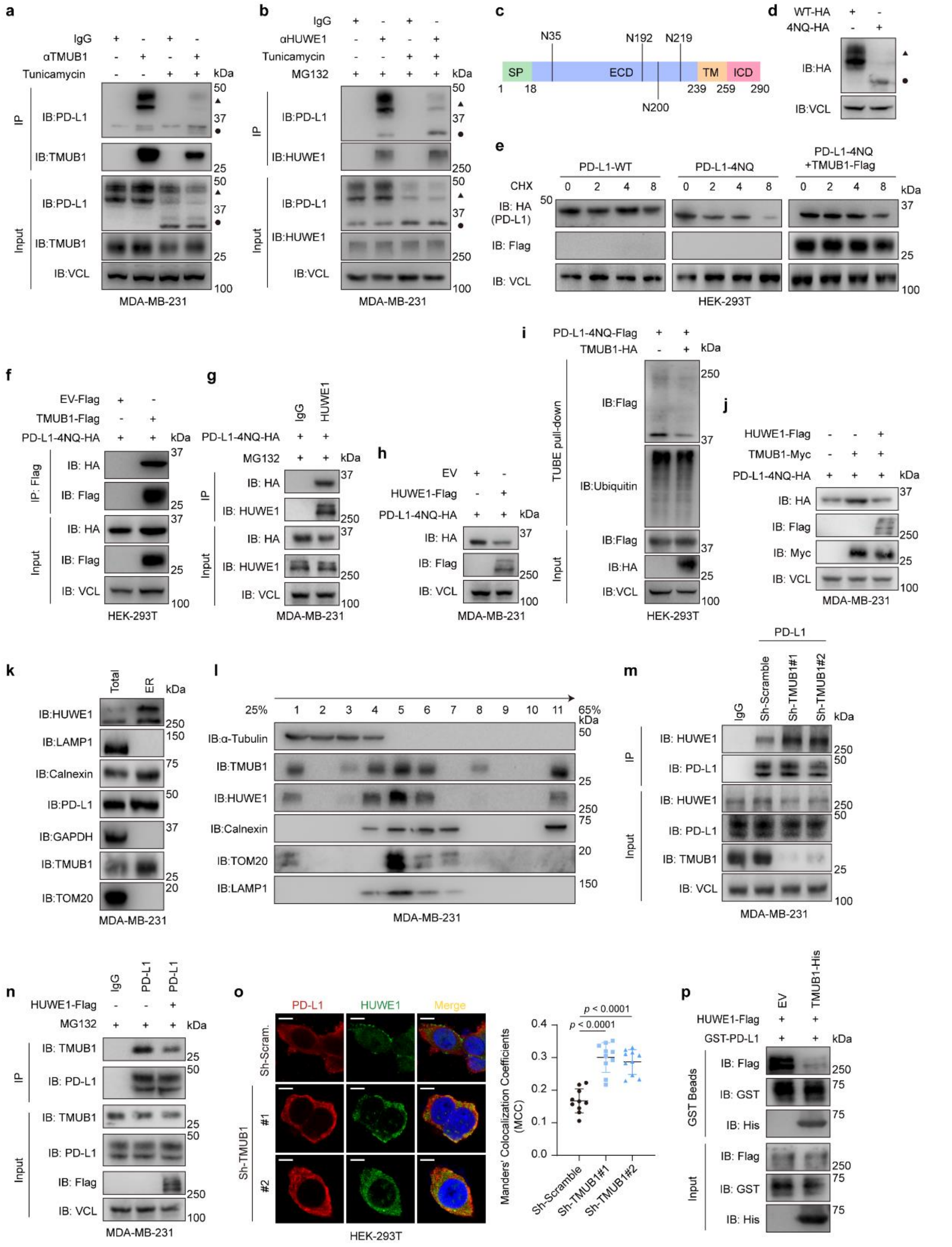

Supplementary Fig. 4, related to Fig. 3. TMUB1 protected PD-L1 from HUWE1-mediated ER-associated degradation

(a-b) Co-IP analysis for the interaction between endogenous PD-L1 and TMUB1 **(a)** or HUWE1 **(b)** within MDA-MB-231 cells treated with 5μg/ml Tunicamycin for 12h.

Triangle: Glycosylated PD-L1, Circle: Non-glycosylated PD-L1. **(c)** The schematic diagram for the N-glycosylation sites on PD-L1. Green: Signal peptide, 1–18; Blue: Extracellular domain, 19–238; Orange: Transmembrane domain, 239–259; Red: Cytoplasmic domain, 260–290; Black bar: the N-glycosylation sites. **(d)** Immunoblots of PD-L1 Wild Type (WT) or 4NQ mutant-HA in HEK-293T cells. Triangle: Glycosylated PD-L1, Circle: Non-glycosylated PD-L1. **(e)** Immunoblots of PD-L1-WT and PD-L1–4NQ with TMUB1 overexpression in HEK-293T cells following treatment with 20 μg/mL of cycloheximide (CHX) for the indicated time durations. **(f)** Co-IP analysis for the interaction between PD-L1–4NQ-HA and TMUB1-Flag in TMUB1-overexpression or control HEK-293T cells. **(g)** Co-IP analysis for the interaction between endogenous HUWE1 and PD-L1-4NQ-HA within MDA-MB-231 cells. **(h)** Immunoblots of PD-L1-4NQ-HA in the MDA-MB-231 cells with HUWE-overexpression or control. **(i)** TUBE pull-down analysis for the interaction between PD-L1-4NQ-Flag and endogenous ubiquitin in TMUB1-overexpression or control HEK-239T cells treated with 10 μM of MG132 for 6 hr. **(j)** Immunoblots of PD-L1-4NQ-HA in the MDA-MB-231 cells with TMUB1 or HUWE1 overexpression. **(k-l)** Analysis of the subcellular localization of PD-L1, TMUB1, and HUWE1 in the fraction of MDA-MB-231 cells isolated by discontinuous sucrose gradient centrifugation **(k)** or continuous sucrose gradient (25%-65%) centrifugation **(l).** Markers for various cell compartments included GAPDH (cytoplasm), LAMP-1 (endocytic compartments), TOM20 (mitochondria), Calnexin (ER). **(m)** Co-IP analysis of the interaction between endogenous HUWE1 and PD-L1 within MDA-MB-231 cells with TMUB1-knockdown

or not. **(n)** Co-IP analysis of the interaction between endogenous TMUB1 and PD-L1 within MDA-MB-231 cells with HUWE1-overexpresion or not. **(o)** Colocalization of PD-L1 and HUWE1 in control or TMUB1-knockdown MDA-MB-231 cells were detected using immunofluorescence staining. Data are presented as mean ± SEM. of n = 10 biologically independent cells. One-way ANOVA followed by Tukey test. **(p)** The GST pull-down assay of the interaction between PD-L1 and HUWE1 with or without TMUB1 was detected using the immunoblot assay.

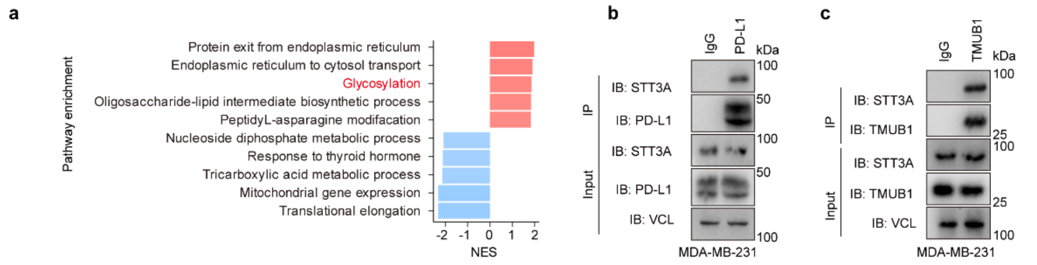

Supplementary Fig. 5, related to Fig. 3. TMUB1 protected PD-L1 from HUWE1-mediated ER-associated degradation

(a) Pathway enrichment was performed by Gene Set Enrichment Analysis (GSEA) using the genes correlated to TMUB1 in **Fig. 3m**. **(b)** Co-IP analysis of the interaction between endogenous PD-L1 and endogenous STT3A within MDA-MB-231 cells. IgG was used as the negative control. **(c)** Co-IP analysis of the interaction between endogenous TMUB1 and endogenous STT3A within MDA-MB-231 cells. IgG was used as the negative control.

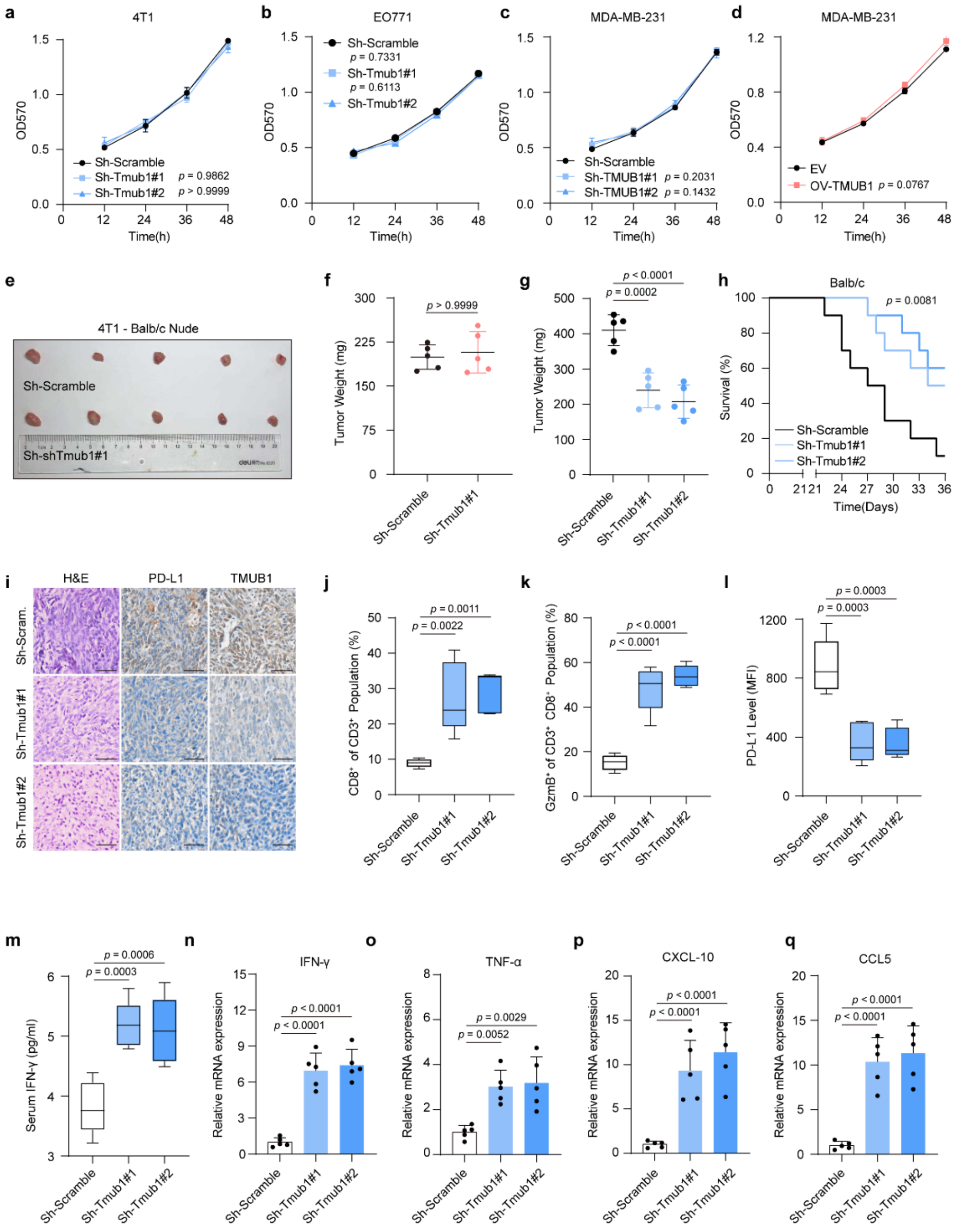

Supplementary Fig. 6, related to Fig. 4. TMUB1-knockdown promoted antitumor immunity *in vivo* via PD-L1 degradation

(a-b) Cell growth viability of control or Tmub1-knockdown 4T1 **(a)** or EO771 **(b)** cells determined using the MTT assay at the indicated time points. Data are presented as mean ± SEM. of n=5. One-way ANOVA followed by Tukey test. **(c-d)** Cell growth viability of TMUB1-knockdown **(c)**, TMUB1-overexpression **(d)** or control MDA-MB-

231 cells determined using the MTT assay at the indicated time points. Data are presented as mean ± SEM. of n = 5. One-way ANOVA followed by Tukey test. **(e)** Xenograft mouse model established using the sh-Scramble or sh-Tmub1 4T1 cells in nude mice (n = 5 mice per group). *In vivo* generated tumors are depicted. **(f)** Analysis of tumor weight in the xenograft nude mouse model. Data are presented as mean ± SEM. of n = 5 mice per group. Two-sided Mann-Whitney test. **(g)** Analysis of tumor weight in the 4T1-Balb/c xenograft mouse model. Data are presented as mean ± SEM. of n = 5 mice per group. One-way ANOVA followed by Tukey test. **(h)** Survival in the mice bearing sh-Scramble or sh-Tmub1 4T1-derived tumor. n = 10 mice per group. Log-rank test. **(i)** Representative IHC staining in randomly selected tumors from mice subcutaneously injected with the indicated stably-transduced 4T1 cells. Scale bar: 100 μm. **(j-m)** Flow cytometric analysis of the $CD3^+$ $CD8^+$ T cells **(j)**, granzyme B-positive $CD3^+$ $CD8^+$ T cells **(k)**, median fluorescence intensity (MFI) of PD-L1 **(l)** and the abundance of IFN-γ (pg/mL) in mice serum in 4T1-Balb/c xenograft mouse model **(m)** was detected by ELISA assays. Data are presented as a box plot with box and whiskers. Bounds of box show the 25th and 75th percentiles, and the central lines in the box represent the median value. Whiskers show min to max value, n = 5 per group. One-way ANOVA followed by Tukey test. **(n-q)** The qRT-PCR analysis of the expressions of IFN-γ **(n),** TNF-α **(o)**, CXCL-10 **(p)**, and CCL-5 **(q)** in bulk 4T1 tumor xenografts. Data presented as mean ± SEM. n = 5. One-way ANOVA followed by Tukey test.

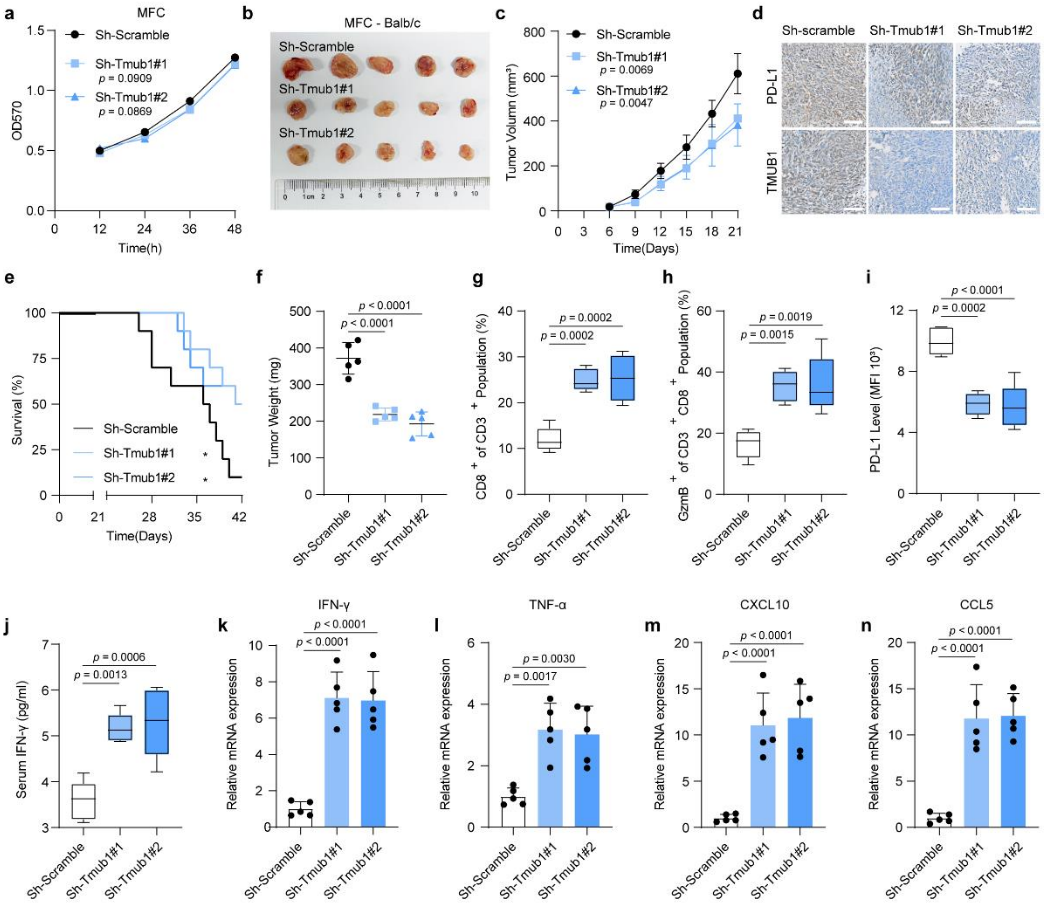

Supplementary Fig. 7, related to Fig. 4. TMUB1-knockdown promoted antitumor immunity *in vivo* via PD-L1 degradation

(a) Cell growth viability of control or Tmub1-knockdown MFC cells determined using the MTT assay at the indicated time points. Data are presented as mean ± SEM. of n=5. One-way ANOVA followed by Tukey test. **(b)** Xenograft mouse model established using the sh-Scramble or sh-Tmub1 MFC cells in Balb/c mice (n = 5 mice per group). *In vivo* generated tumors are depicted. **(c)** Analysis of tumor growth and volume in the xenograft mouse model. Data are presented as mean ± SEM. of n = 5 mice per group. Two-way ANOVA. **(d)** Representative IHC staining in randomly selected tumors from mice subcutaneously injected with the indicated stably-transduced MFC cells. Scale bar:

100 μm. **(e)** Survival in the mice bearing sh-Scramble or sh-Tmub1 MFC-derived tumor. n = 10 mice per group. Log-rank test. **(f)** Analysis of tumor weight in the xenograft mouse model. Data are presented as mean ± SEM. of n = 5 mice per group. One-way ANOVA followed by Tukey test. **(g-j)** Flow cytometric analysis of the $CD3^{+}$ $CD8^{+}$ T cells **(g)**, granzyme B-positive $CD3^{+}$ $CD8^{+}$ T cells **(h)**, median fluorescence intensity (MFI) of PD-L1 **(i)** and the abundance of IFN-γ (pg/mL) in mice serum **(j)** was detected by ELISA assays in MFC-Balb/c xenograft mouse model. Data are presented as mean ± SEM. of n = 5 per group. One-way ANOVA followed by Tukey test. **(k-n)** The qRT-PCR analysis of the expressions of IFN-γ **(k),** TNF-α **(l)**, CXCL-10 **(m)**, and CCL-5 **(n)** in bulk MFC tumor xenografts. Data presented as mean ± SEM. n = 5. One-way ANOVA followed by Tukey test.

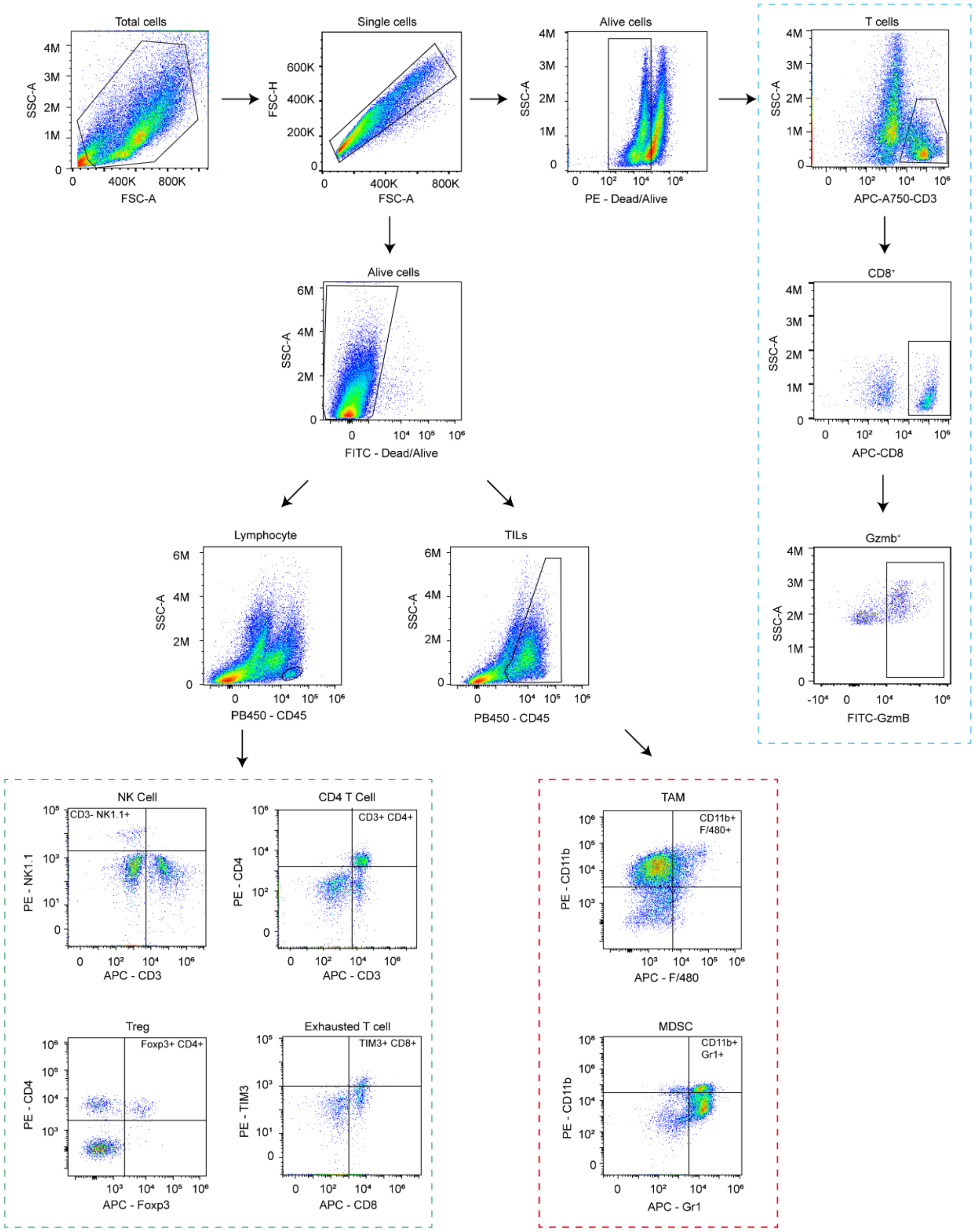

Supplementary Fig. 8, FACS gating strategy for detection of immune cell in TILs.

Live cells in leukocyte fraction were gated via FSC and SSC. After exclusion of dead cells. In the blue dashed box: T-cell population was gated based on the surface expression of CD3. $CD8^+$ CTL population was gated based on surface expression of CD8. Activation of CTL was evaluated by expression of granzyme B. In the green

dashed box: Leukocytes population was gated based on the surface expression of CD45. NK cells population was gated based on the expression of CD3 and NK1.1. CD4 T cells population was gated based on the expression of CD3 and CD4. Treg cells population was gated based on the expression of Foxp3 and CD4. Exhausted T cell population was gated based on the expression of CD8 and TIM3. In the red dashed box: Leukocytes population was gated based on the surface expression of CD45. TAM cells population was gated based on the surface expression of CD11b and F/480. MDSC cells population was gated based on the surface expression of CD11b and Gr1. Quantitation shown in **Figure 4j-r, 7h-i, 7n-o** and **Supplementary Figure 6j-k, 7g-h, 11g-h, 12f-g, 13a-f**.

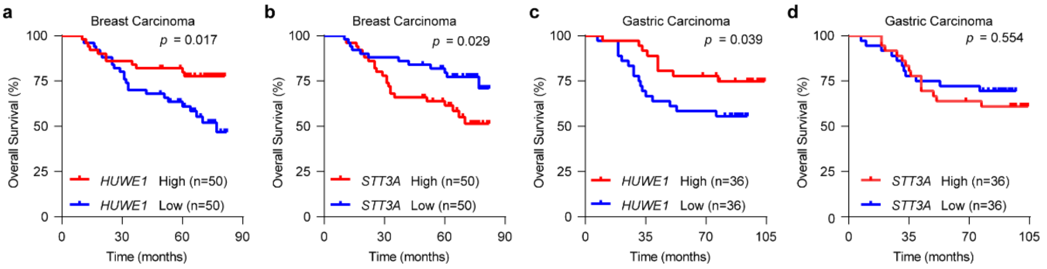

Supplementary Fig. 9, related to Fig. 5. Clinical value of TMUB1 as a potential target in tumor immunotherapy.

(a-b) Kaplan-Meier analysis of the overall survival curve for breast cancer patients (Sun Yat-sen cohorts; n = 100) with low or high HUWE1 **(a)** or STT3A **(b)** expression. The RNA levels were detected using qRT-PCR relative to GAPDH. Kaplan-Meier analysis along with the log-rank test. **(c-d)** Kaplan-Meier analysis of the overall survival curve for gastric cancer patients (Second Affiliated Hospital, School of Medicine Zhejiang University cohorts; n = 72) with low or high HUWE1 **(c)** or STT3A **(d)** expression. The RNA levels were detected using qRT-PCR relative to GAPDH. Kaplan-Meier analysis along with the log-rank test.

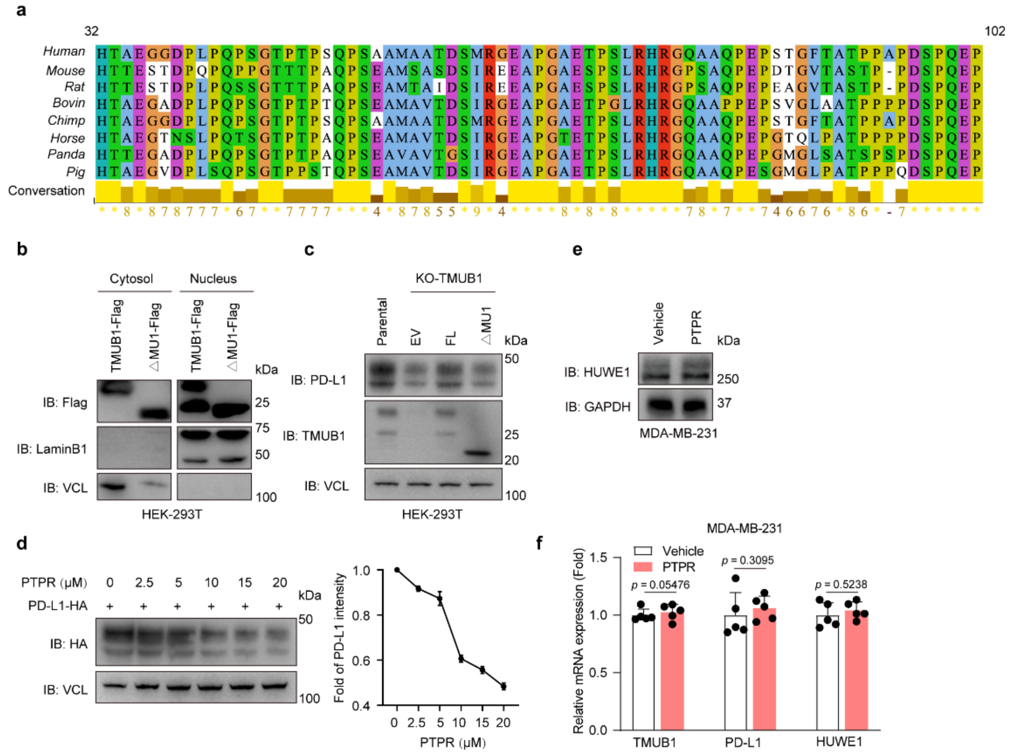

Supplementary Fig. 10, related to Fig. 6. The competitive peptide PTPR weakened the upregulation of PD-L1 caused by TMUB1

(a) Homology analysis of the MU1 domain of TMUB1 in mammals. **(b)** Subcellular localization of TMUB1-FL-Flag and TMUB1-ΔMU1-Flag in HEK-293T cells. **(c)** Immunoblots of PD-L1 in TMUB1-FL and TMUB1-ΔMU1-rescued HEK-293T TMUB1-KO cells. **(d)** Immunoblots of PD-L1 or in the MDA-MB-231 cells treated with a gradient (increasing concentrations 0 to 20 μM) of PTPR. Data presented as mean ± SEM. n = 3. **(e)** Immunoblots of HUWE1 in the MDA-MB-231 cells treated with 10 μM of PTPR for 12 hr or not. **(f)** The qRT-PCR analysis of the mRNA expression of PD-L1/TMUIB1/HUWE1 in the MDA-MB-231 cells treated with 10 μM of PTPR for 12 hr or not. Data presented as mean ± SEM. Two-sided Mann-Whitney test.

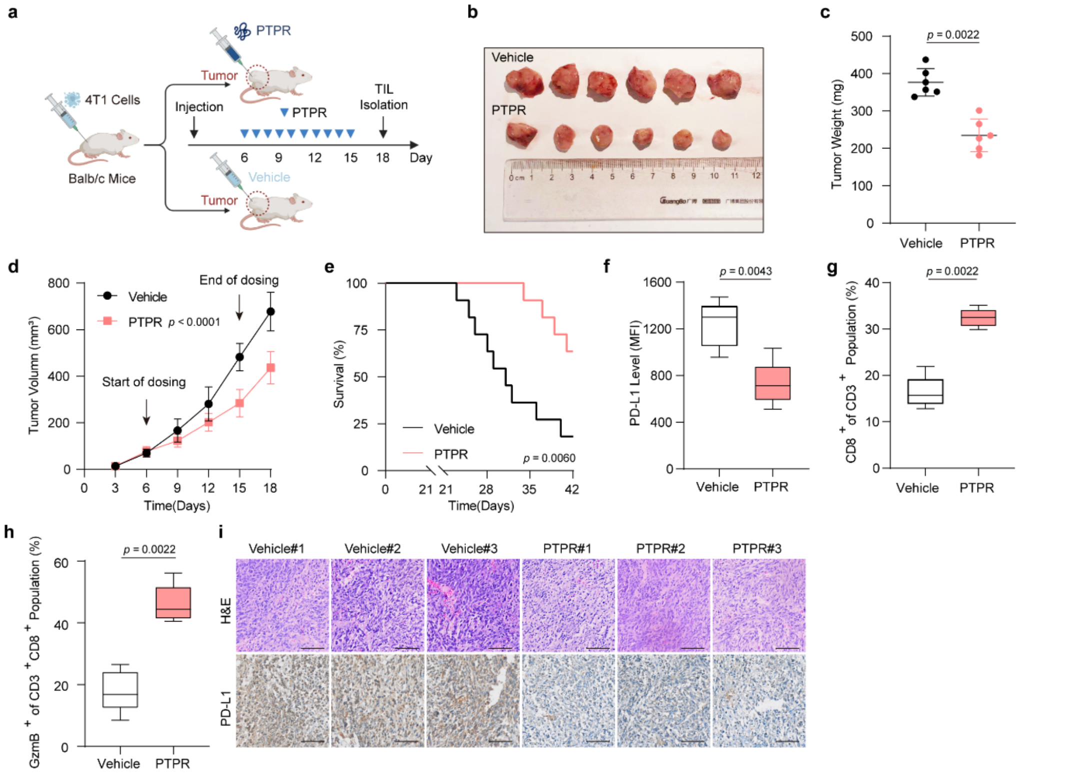

Supplementary Fig. 11, related to Fig. 7. *In vivo* antitumor effect and toxicity of PTPR

(a) The injection schematic for PTPR and *in vivo* antitumor effect analysis using 4T1 cells. **(b)** Xenograft mouse model established using 4T1 cells in Balb/c mice injected with PTPR or Vehicle (n = 6 mice per group). *In vivo* generated tumors are depicted. **(c)** Analysis of tumor weight in the xenograft mouse model. Data are presented as mean ± SEM. of n = 6 mice per group. Two-sided Mann-Whitney test. **(d)** Analysis of tumor growth and volume in the xenograft mouse model. Data are presented as mean ± SEM of n = 6 mice per group. Two-way ANOVA. **(e)** Survival in the mice injected with PTPR or vehicle. n = 11 mice per group. Log-rank test. **(f-h)** Flow cytometric analysis of median fluorescence intensity (MFI) of PD-L1 **(f)**, $CD3^+$ $CD8^+$ T cells **(g)** and granzyme B-positive $CD3^+$ $CD8^+$ T cells **(h)** in mice treated with PTPR or vehicle. Data are

presented as a box plot with box and whiskers. Bounds of box show the 25th and 75th percentiles, and the central lines in the box represent the median value. Whiskers show min to max value, n = 6 per group. Two-sided Mann-Whitney test. **(i)** Representative IHC staining in randomly selected tumors from mice treated with PTPR or vehicle. Scale bar: 100 μm.

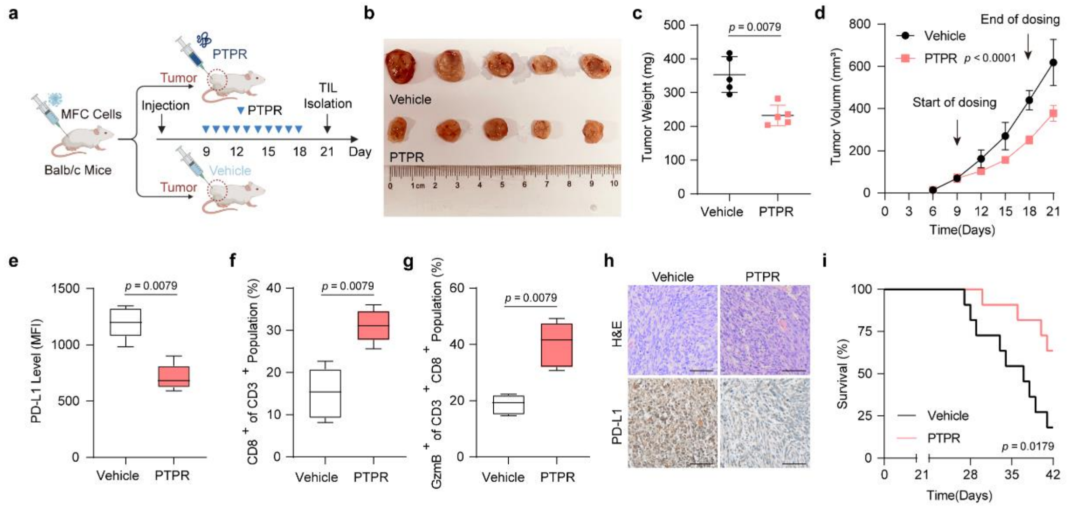

Supplementary Fig. 12, related to Fig. 7. ***In vivo*** **antitumor effect and toxicity of PTPR**

(a) The injection schematic for PTPR and *in vivo* antitumor effect analysis using MFC cells. **(b)** Xenograft mouse model established using MFC cells in Balb/c mice injected with PTPR or Vehicle (n = 5 mice per group). *In vivo* generated tumors are depicted. **(c)** Analysis of tumor weight in the xenograft mouse model. Data are presented as mean ± SEM. of n = 5 mice per group. Two-sided Mann-Whitney test. **(d)** Analysis of tumor growth and volume in the xenograft mouse model. Data are presented as mean ± SEM of n = 5 mice per group. Two-way ANOVA. **(e-g)** Flow cytometric analysis with median fluorescence intensity (MFI) of PD-L1 **(e)**, $CD3^+$ $CD8^+$ T cells **(f)** and granzyme B-positive $CD3^+$ $CD8^+$ T cells **(g)** in mice treated with PTPR or vehicle. Data are presented as a box plot with box and whiskers. Bounds of box show the 25th and 75th percentiles, and the central lines in the box represent the median value. Whiskers show min to max value, n = 5 per group. Two-sided Mann-Whitney test. **(h)** Representative IHC staining in randomly selected tumors. Scale bar: 100 µm. **(i)** Survival in the mice injected with PTPR or vehicle. n = 10 mice per group. Log-rank test.

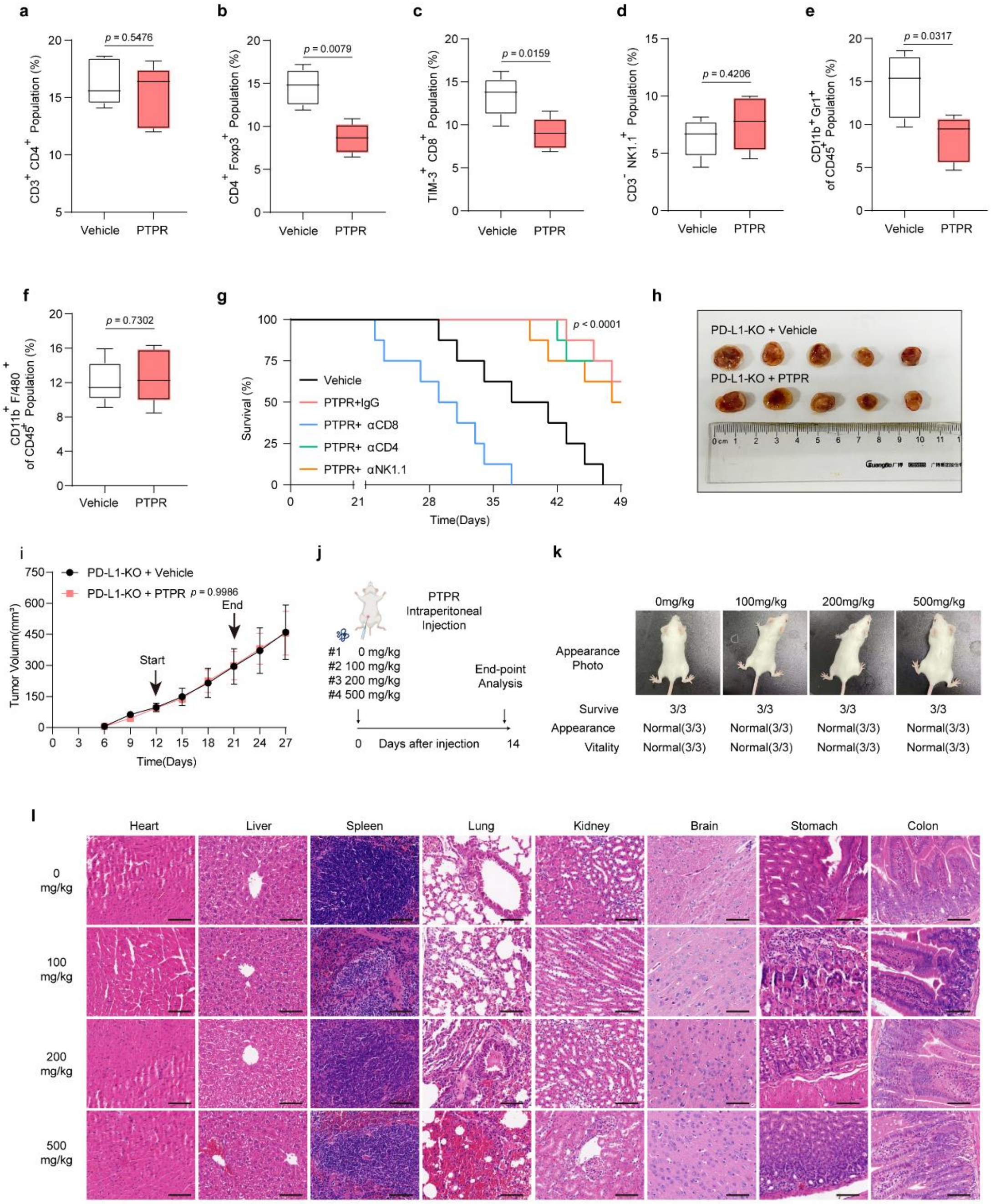

Supplementary Fig. 13, related to Fig. 7. ***In vivo*** **antitumor effect and toxicity of PTPR**

(a-f) Flow cytometric analysis of the CD3$^+$ CD4$^+$ T cells **(a)**, CD4$^+$ Foxp3$^+$ Treg cells **(b)**, TIM3$^+$ CD8$^+$ $T_{Exhausted}$ cells **(c)**, CD3$^-$ NK1.1$^+$ NK cells **(d)**, CD11b$^+$ Gr1$^+$ MDSC **(e)** and CD11b$^+$ F/480$^+$ TAM cells **(f)** in EO771 tumor in mice treated with PTPR or vehicle. Data are presented as a box plot with box and whiskers. Bounds of box show

the 25th and 75th percentiles, and the central lines in the box represent the median value. Whiskers show min to max value, n = 5 per group. Two-sided Mann-Whitney test. **(g)** Survival in the mice with indicated treatment. n = 10 mice per group. Log-rank test. **(h)** Xenograft mouse model established using PD-L1-KO 4T1 cells in Balb/c mice injected with PTPR or Vehicle (n = 5 mice per group). In vivo generated tumors are depicted. **(i)** Analysis of tumor growth and volume in the xenograft mouse model. Data are presented as mean ± SEM. of n = 5 mice per group. Two-way ANOVA. **(j)** The injection schematic for PTPR and *in vivo* toxicity analysis **(k)** The survival, gross appearance, and vitality of mice at the endpoint of observation. In each group, three mice were observed on gross appearance and vitality. **(l)** The H&E staining of tissue slices from different organs of mice treated with different doses of PTPR. Scale bar: 100 µm.

Supplementary Table 1. Protein Identification Results for PD-L1-Flag IP assay

Accession	Description	PD-L1-Flag (PSMs)	EV-Flag (PSMs)	Foldchange PD-L1/EV
Q9NZQ7	Programmed cell death 1 ligand 1 OS=Homo sapiens OX=9606 GN=CD274 PE=1 SV=1	114	5	22.80
P55060	Exportin-2 OS=Homo sapiens OX=9606 GN=CSE1L PE=1 SV=3	19	1	19.00
O95373	Importin-7 OS=Homo sapiens OX=9606 GN=IPO7 PE=1 SV=1	18	1	18.00
Q92616	eIF-2-alpha kinase activator GCN1 OS=Homo sapiens OX=9606 GN=GCN1 PE=1 SV=6	17	1	17.00
O00410	Importin-5 OS=Homo sapiens OX=9606 GN=IPO5 PE=1 SV=4	13	1	13.00
O14980	Exportin-1 OS=Homo sapiens OX=9606 GN=XPO1 PE=1 SV=1	23	2	11.50
Q86VP6	Cullin-associated NEDD8-dissociated protein 1 OS=Homo sapiens OX=9606 GN=CAND1 PE=1 SV=2	10	1	10.00
Q14008	Cytoskeleton-associated protein 5 OS=Homo sapiens OX=9606 GN=CKAP5 PE=1 SV=3	9	1	9.00
Q13085	Acetyl-CoA carboxylase 1 OS=Homo sapiens OX=9606 GN=ACACA PE=1 SV=2	8	1	8.00
P42704	Leucine-rich PPR motif-containing protein, mitochondrial OS=Homo sapiens OX=9606 GN=LRPPRC PE=1 SV=3	8	1	8.00
Q6P3W7	SCY1-like protein 2 OS=Homo sapiens OX=9606 GN=SCYL2 PE=1 SV=1	6	1	6.00
O00203	AP-3 complex subunit beta-1 OS=Homo sapiens OX=9606 GN=AP3B1 PE=1 SV=3	6	1	6.00
Q14974	Importin subunit beta-1 OS=Homo sapiens OX=9606 GN=KPNB1 PE=1 SV=2	10	2	5.00
Q7Z6Z7	E3 ubiquitin-protein ligase HUWE1 OS=Homo sapiens OX=9606 GN=HUWE1 PE=1 SV=3	10	2	5.00
O00571	ATP-dependent RNA helicase DDX3X OS=Homo sapiens OX=9606 GN=DDX3X PE=1 SV=3	5	1	5.00
P33991	DNA replication licensing factor MCM4 OS=Homo sapiens OX=9606 GN=MCM4 PE=1 SV=5	5	1	5.00
Q9NVI7	ATPase family AAA domain-containing protein 3A OS=Homo sapiens OX=9606 GN=ATAD3A PE=1 SV=2	5	1	5.00
O95831	Apoptosis-inducing factor 1, mitochondrial OS=Homo sapiens OX=9606 GN=AIFM1 PE=1 SV=1	5	1	5.00
Q14566	DNA replication licensing factor MCM6 OS=Homo sapiens OX=9606 GN=MCM6 PE=1 SV=1	5	1	5.00
P57740	Nuclear pore complex protein Nup107 OS=Homo sapiens OX=9606 GN=NUP107 PE=1 SV=1	9	2	4.50
P05023	Sodium/potassium-transporting ATPase subunit alpha-1 OS=Homo sapiens OX=9606 GN=ATP1A1 PE=1 SV=1	13	3	4.33
Q15750	TGF-beta-activated kinase 1 and MAP3K7-binding protein 1 OS=Homo sapiens OX=9606 GN=TAB1 PE=1 SV=1	17	4	4.25
Q9Y678	Coatomer subunit gamma-1 OS=Homo sapiens OX=9606 GN=COPG1 PE=1 SV=1	12	3	4.00
O60271	C-Jun-amino-terminal kinase-interacting protein 4 OS=Homo sapiens OX=9606 GN=SPAG9 PE=1 SV=4	4	1	4.00
P07814	Bifunctional glutamate/proline--tRNA ligase OS=Homo sapiens OX=9606 GN=EPRS PE=1 SV=5	4	1	4.00
Q9NTJ3	Structural maintenance of chromosomes protein 4 OS=Homo sapiens OX=9606 GN=SMC4 PE=1 SV=2	4	1	4.00
Q14739	Lamin-B receptor OS=Homo sapiens OX=9606 GN=LBR PE=1 SV=2	4	1	4.00
P11586	C-1-tetrahydrofolate synthase, cytoplasmic OS=Homo sapiens OX=9606 GN=MTHFD1 PE=1 SV=3	4	1	4.00
P25789	Proteasome subunit alpha type-4 OS=Homo sapiens OX=9606 GN=PSMA4 PE=1 SV=1	4	1	4.00
O75369	Filamin-B OS=Homo sapiens OX=9606 GN=FLNB PE=1 SV=2	15	4	3.75
P16615	Sarcoplasmic/endoplasmic reticulum calcium ATPase 2 OS=Homo sapiens OX=9606 GN=ATP2A2 PE=1 SV=1	18	5	3.60
O95347	Structural maintenance of chromosomes protein 2 OS=Homo sapiens OX=9606 GN=SMC2 PE=1 SV=2	7	2	3.50
P42356	Phosphatidylinositol 4-kinase alpha OS=Homo sapiens OX=9606 GN=PI4KA PE=1 SV=4	7	2	3.50
P33993	DNA replication licensing factor MCM7 OS=Homo sapiens OX=9606 GN=MCM7 PE=1 SV=4	7	2	3.50
Q99832	T-complex protein 1 subunit eta OS=Homo sapiens OX=9606 GN=CCT7 PE=1 SV=2	22	7	3.14
P04843	Dolichyl-diphosphooligosaccharide--protein glycosyltransferase subunit 1 OS=Homo sapiens OX=9606 GN=RPN1 PE=1 SV=1	15	5	3.00
P53618	Coatomer subunit beta OS=Homo sapiens OX=9606 GN=COPB1 PE=1 SV=3	12	4	3.00
P33176	Kinesin-1 heavy chain OS=Homo sapiens OX=9606 GN=KIF5B PE=1 SV=1	9	3	3.00
Q96N67	Dedicator of cytokinesis protein 7 OS=Homo sapiens OX=9606 GN=DOCK7 PE=1 SV=4	9	3	3.00
Q6Y7W6	GRB10-interacting GYF protein 2 OS=Homo sapiens OX=9606 GN=GIGYF2 PE=1 SV=1	6	2	3.00
Q99460	26S proteasome non-ATPase regulatory subunit 1 OS=Homo sapiens OX=9606 GN=PSMD1 PE=1 SV=2	6	2	3.00
Q16531	DNA damage-binding protein 1 OS=Homo sapiens OX=9606 GN=DDB1 PE=1 SV=1	6	2	3.00
Q92945	Far upstream element-binding protein 2 OS=Homo sapiens OX=9606 GN=KHSRP PE=1 SV=4	3	1	3.00
P62258	14-3-3 protein epsilon OS=Homo sapiens OX=9606 GN=YWHAE PE=1 SV=1	3	1	3.00
O15371	Eukaryotic translation initiation factor 3 subunit D OS=Homo sapiens OX=9606 GN=EIF3D PE=1 SV=1	3	1	3.00
Q9UHD8	Septin-9 OS=Homo sapiens OX=9606 GN=SEPT9 PE=1 SV=2	3	1	3.00
P52292	Importin subunit alpha-1 OS=Homo sapiens OX=9606 GN=KPNA2 PE=1 SV=1	3	1	3.00
Q8N1F7	Nuclear pore complex protein Nup93 OS=Homo sapiens OX=9606 GN=NUP93 PE=1 SV=2	3	1	3.00
P20700	Lamin-B1 OS=Homo sapiens OX=9606 GN=LMNB1 PE=1 SV=2	3	1	3.00
Q07065	Cytoskeleton-associated protein 4 OS=Homo sapiens OX=9606 GN=CKAP4 PE=1 SV=2	11	4	2.75
P68371	Tubulin beta-4B chain OS=Homo sapiens OX=9606 GN=TUBB4B PE=1 SV=1	23	9	2.56
P53396	ATP-citrate synthase OS=Homo sapiens OX=9606 GN=ACLY PE=1 SV=3	15	6	2.50
P46379	Large proline-rich protein BAG6 OS=Homo sapiens OX=9606 GN=BAG6 PE=1 SV=2	5	2	2.50
Q5U651	Ras-interacting protein 1 OS=Homo sapiens OX=9606 GN=RASIP1 PE=1 SV=1	5	2	2.50
O15397	Importin-8 OS=Homo sapiens OX=9606 GN=IPO8 PE=1 SV=2	26	11	2.36
Q9UQE7	Structural maintenance of chromosomes protein 3 OS=Homo sapiens OX=9606 GN=SMC3 PE=1 SV=2	7	3	2.33
P40227	T-complex protein 1 subunit zeta OS=Homo sapiens OX=9606 GN=CCT6A PE=1 SV=3	16	7	2.29
P35606	Coatomer subunit beta' OS=Homo sapiens OX=9606 GN=COPB2 PE=1 SV=2	9	4	2.25
Q9Y657	Spindlin-1 OS=Homo sapiens OX=9606 GN=SPIN1 PE=1 SV=3	9	4	2.25
O00268	Transcription initiation factor TFIID subunit 4 OS=Homo sapiens OX=9606 GN=TAF4 PE=1 SV=2	9	4	2.25
Q9NXV2	BTB/POZ domain-containing protein KCTD5 OS=Homo sapiens OX=9606 GN=KCTD5 PE=1 SV=1	9	4	2.25
P78527	DNA-dependent protein kinase catalytic subunit OS=Homo sapiens OX=9606 GN=PRKDC PE=1 SV=3	49	22	2.23
P53621	Coatomer subunit alpha OS=Homo sapiens OX=9606 GN=COPA PE=1 SV=2	22	10	2.20
P68363	Tubulin alpha-1B chain OS=Homo sapiens OX=9606 GN=TUBA1B PE=1 SV=1	28	13	2.15
P49327	Fatty acid synthase OS=Homo sapiens OX=9606 GN=FASN PE=1 SV=3	57	27	2.11
P23458	Tyrosine-protein kinase JAK1 OS=Homo sapiens OX=9606 GN=JAK1 PE=1 SV=2	25	12	2.08
P07437	Tubulin beta chain OS=Homo sapiens OX=9606 GN=TUBB PE=1 SV=2	26	13	2.00
O75643	U5 small nuclear ribonucleoprotein 200 kDa helicase OS=Homo sapiens OX=9606 GN=SNRNP200 PE=1 SV=2	12	6	2.00
Q9P2R3	Rabankyrin-5 OS=Homo sapiens OX=9606 GN=ANKFY1 PE=1 SV=2	12	6	2.00
Q9BVT8	Transmembrane and ubiquitin-like domain-containing protein 1 OS=Homo sapiens OX=9606 GN=TMUB1 PE=1 SV=1	10	5	2.00
Q16891	MICOS complex subunit MIC60 OS=Homo sapiens OX=9606 GN=IMMT PE=1 SV=1	8	4	2.00
Q8TEM1	Nuclear pore membrane glycoprotein 210 OS=Homo sapiens OX=9606 GN=NUP210 PE=1 SV=3	6	3	2.00
Q9UJS0	Calcium-binding mitochondrial carrier protein Aralar2 OS=Homo sapiens OX=9606 GN=SLC25A13 PE=1 SV=2	4	2	2.00
P23396	40S ribosomal protein S3 OS=Homo sapiens OX=9606 GN=RPS3 PE=1 SV=2	4	2	2.00
P54886	Delta-1-pyrroline-5-carboxylate synthase OS=Homo sapiens OX=9606 GN=ALDH18A1 PE=1 SV=2	4	2	2.00
Q04637	Eukaryotic translation initiation factor 4 gamma 1 OS=Homo sapiens OX=9606 GN=EIF4G1 PE=1 SV=4	4	2	2.00
Q9P1Y5	Calmodulin-regulated spectrin-associated protein 3 OS=Homo sapiens OX=9606 GN=CAMSAP3 PE=1 SV=2	4	2	2.00
O94776	Metastasis-associated protein MTA2 OS=Homo sapiens OX=9606 GN=MTA2 PE=1 SV=1	2	1	2.00
Q12769	Nuclear pore complex protein Nup160 OS=Homo sapiens OX=9606 GN=NUP160 PE=1 SV=3	2	1	2.00
P49720	Proteasome subunit beta type-3 OS=Homo sapiens OX=9606 GN=PSMB3 PE=1 SV=2	2	1	2.00
Q9NYJ8	TGF-beta-activated kinase 1 and MAP3K7-binding protein 2 OS=Homo sapiens OX=9606 GN=TAB2 PE=1 SV=1	2	1	2.00
P35998	26S proteasome regulatory subunit 7 OS=Homo sapiens OX=9606 GN=PSMC2 PE=1 SV=3	2	1	2.00
Q9C0C9	(E3-independent) E2 ubiquitin-conjugating enzyme OS=Homo sapiens OX=9606 GN=UBE2O PE=1 SV=3	2	1	2.00
Q9Y230	RuvB-like 2 OS=Homo sapiens OX=9606 GN=RUVBL2 PE=1 SV=3	2	1	2.00
Q9Y285	Phenylalanine--tRNA ligase alpha subunit OS=Homo sapiens OX=9606 GN=FARSA PE=1 SV=3	2	1	2.00
Q15393	Splicing factor 3B subunit 3 OS=Homo sapiens OX=9606 GN=SF3B3 PE=1 SV=4	2	1	2.00
P30153	Serine/threonine-protein phosphatase 2A 65 kDa regulatory subunit A alpha isoform OS=Homo sapiens OX=9606 GN=PPP2R1A PE=1 SV=4	2	1	2.00
Q9BPX3	Condensin complex subunit 3 OS=Homo sapiens OX=9606 GN=NCAPG PE=1 SV=1	2	1	2.00
Q5SWX8	Protein odr-4 homolog OS=Homo sapiens OX=9606 GN=ODR4 PE=1 SV=1	2	1	2.00
P60228	Eukaryotic translation initiation factor 3 subunit E OS=Homo sapiens OX=9606 GN=EIF3E PE=1 SV=1	2	1	2.00
Q13310	Polyadenylate-binding protein 4 OS=Homo sapiens OX=9606 GN=PABPC4 PE=1 SV=1	2	1	2.00
O14579	Coatomer subunit epsilon OS=Homo sapiens OX=9606 GN=COPE PE=1 SV=3	2	1	2.00
P04844	Dolichyl-diphosphooligosaccharide--protein glycosyltransferase subunit 2 OS=Homo sapiens OX=9606 GN=RPN2 PE=1 SV=3	2	1	2.00
P02545	Prelamin-A/C OS=Homo sapiens OX=9606 GN=LMNA PE=1 SV=1	2	1	2.00
O75489	NADH dehydrogenase [ubiquinone] iron-sulfur protein 3, mitochondrial OS=Homo sapiens OX=9606 GN=NDUFS3 PE=1 SV=1	2	1	2.00
P39656	Dolichyl-diphosphooligosaccharide--protein glycosyltransferase 48 kDa subunit OS=Homo sapiens OX=9606 GN=DDOST PE=1 SV=4	2	1	2.00
Q4VCS5	Angiomotin OS=Homo sapiens OX=9606 GN=AMOT PE=1 SV=1	37	19	1.95
P05141	ADP/ATP translocase 2 OS=Homo sapiens OX=9606 GN=SLC25A5 PE=1 SV=7	13	7	1.86
P0DMV8	Heat shock 70 kDa protein 1A OS=Homo sapiens OX=9606 GN=HSPA1A PE=1 SV=1	36	20	1.80
P14625	Endoplasmin OS=Homo sapiens OX=9606 GN=HSP90B1 PE=1 SV=1	18	10	1.80
P25205	DNA replication licensing factor MCM3 OS=Homo sapiens OX=9606 GN=MCM3 PE=1 SV=3	9	5	1.80
Q15029	116 kDa U5 small nuclear ribonucleoprotein component OS=Homo sapiens OX=9606 GN=EFTUD2 PE=1 SV=1	9	5	1.80
Q99623	Prohibitin-2 OS=Homo sapiens OX=9606 GN=PHB2 PE=1 SV=2	9	5	1.80
P11021	Endoplasmic reticulum chaperone BiP OS=Homo sapiens OX=9606 GN=HSPA5 PE=1 SV=2	50	28	1.79
P50991	T-complex protein 1 subunit delta OS=Homo sapiens OX=9606 GN=CCT4 PE=1 SV=4	16	9	1.78
Q92598	Heat shock protein 105 kDa OS=Homo sapiens OX=9606 GN=HSPH1 PE=1 SV=1	7	4	1.75
Q13464	Rho-associated protein kinase 1 OS=Homo sapiens OX=9606 GN=ROCK1 PE=1 SV=1	15	9	1.67
P27708	CAD protein OS=Homo sapiens OX=9606 GN=CAD PE=1 SV=3	10	6	1.67
P38646	Stress-70 protein, mitochondrial OS=Homo sapiens OX=9606 GN=HSPA9 PE=1 SV=2	21	13	1.62
P21333	Filamin-A OS=Homo sapiens OX=9606 GN=FLNA PE=1 SV=4	50	31	1.61
P48643	T-complex protein 1 subunit epsilon OS=Homo sapiens OX=9606 GN=CCT5 PE=1 SV=1	11	7	1.57
P55072	Transitional endoplasmic reticulum ATPase OS=Homo sapiens OX=9606 GN=VCP PE=1 SV=4	49	32	1.53
P08238	Heat shock protein HSP 90-beta OS=Homo sapiens OX=9606 GN=HSP90AB1 PE=1 SV=4	26	17	1.53
P23588	Eukaryotic translation initiation factor 4B OS=Homo sapiens OX=9606 GN=EIF4B PE=1 SV=2	21	14	1.50
P46459	Vesicle-fusing ATPase OS=Homo sapiens OX=9606 GN=NSF PE=1 SV=3	6	4	1.50
P01040	Cystatin-A OS=Homo sapiens OX=9606 GN=CSTA PE=1 SV=1	3	2	1.50
P24928	DNA-directed RNA polymerase II subunit RPB1 OS=Homo sapiens OX=9606 GN=POLR2A PE=1 SV=2	3	2	1.50
P06733	Alpha-enolase OS=Homo sapiens OX=9606 GN=ENO1 PE=1 SV=2	3	2	1.50
Q9UBF2	Coatomer subunit gamma-2 OS=Homo sapiens OX=9606 GN=COPG2 PE=1 SV=1	3	2	1.50
P61978	Heterogeneous nuclear ribonucleoprotein K OS=Homo sapiens OX=9606 GN=HNRNPK PE=1 SV=1	3	2	1.50
O95163	Elongator complex protein 1 OS=Homo sapiens OX=9606 GN=ELP1 PE=1 SV=3	3	2	1.50
P01023	Alpha-2-macroglobulin OS=Homo sapiens OX=9606 GN=A2M PE=1 SV=3	3	2	1.50
A6NHR9	Structural maintenance of chromosomes flexible hinge domain-containing protein 1 OS=Homo sapiens OX=9606 GN=SMCHD1 PE=1 SV=2	3	2	1.50
P33992	DNA replication licensing factor MCM5 OS=Homo sapiens OX=9606 GN=MCM5 PE=1 SV=5	3	2	1.50
P52948	Nuclear pore complex protein Nup98-Nup96 OS=Homo sapiens OX=9606 GN=NUP98 PE=1 SV=4	3	2	1.50
P49368	T-complex protein 1 subunit gamma OS=Homo sapiens OX=9606 GN=CCT3 PE=1 SV=4	22	15	1.47
P17987	T-complex protein 1 subunit alpha OS=Homo sapiens OX=9606 GN=TCP1 PE=1 SV=1	20	14	1.43
P50990	T-complex protein 1 subunit theta OS=Homo sapiens OX=9606 GN=CCT8 PE=1 SV=4	17	12	1.42
P49736	DNA replication licensing factor MCM2 OS=Homo sapiens OX=9606 GN=MCM2 PE=1 SV=4	7	5	1.40

P25705	ATP synthase subunit alpha, mitochondrial OS=Homo sapiens OX=9606 GN=ATP5F1A PE=1 SV=1	18	13	1.38
P78371	T-complex protein 1 subunit beta OS=Homo sapiens OX=9606 GN=CCT2 PE=1 SV=4	27	20	1.35
P11142	Heat shock cognate 71 kDa protein OS=Homo sapiens OX=9606 GN=HSPA8 PE=1 SV=1	35	26	1.35
P62979	Ubiquitin-40S ribosomal protein S27a OS=Homo sapiens OX=9606 GN=RPS27A PE=1 SV=2	8	6	1.33
P48444	Coatomer subunit delta OS=Homo sapiens OX=9606 GN=ARCN1 PE=1 SV=1	4	3	1.33
O14818	Proteasome subunit alpha type-7 OS=Homo sapiens OX=9606 GN=PSMA7 PE=1 SV=1	4	3	1.33
P54105	Methylosome subunit pICln OS=Homo sapiens OX=9606 GN=CLNS1A PE=1 SV=1	4	3	1.33
Q9Y2H1	Serine/threonine-protein kinase 38-like OS=Homo sapiens OX=9606 GN=STK38L PE=1 SV=3	29	22	1.32
P55884	Eukaryotic translation initiation factor 3 subunit B OS=Homo sapiens OX=9606 GN=EIF3B PE=1 SV=3	14	11	1.27
Q15208	Serine/threonine-protein kinase 38 OS=Homo sapiens OX=9606 GN=STK38 PE=1 SV=1	55	44	1.25
Q9Y262	Eukaryotic translation initiation factor 3 subunit L OS=Homo sapiens OX=9606 GN=EIF3L PE=1 SV=1	10	8	1.25
P26640	Valine--tRNA ligase OS=Homo sapiens OX=9606 GN=VARS PE=1 SV=4	5	4	1.25
P49902	Cytosolic purine 5'-nucleotidase OS=Homo sapiens OX=9606 GN=NT5C2 PE=1 SV=1	5	4	1.25
P35232	Prohibitin OS=Homo sapiens OX=9606 GN=PHB PE=1 SV=1	5	4	1.25
Q6P2Q9	Pre-mRNA-processing-splicing factor 8 OS=Homo sapiens OX=9606 GN=PRPF8 PE=1 SV=2	11	9	1.22
P68104	Elongation factor 1-alpha 1 OS=Homo sapiens OX=9606 GN=EEF1A1 PE=1 SV=1	13	11	1.18
P10809	60 kDa heat shock protein, mitochondrial OS=Homo sapiens OX=9606 GN=HSPD1 PE=1 SV=2	13	11	1.18
O43242	26S proteasome non-ATPase regulatory subunit 3 OS=Homo sapiens OX=9606 GN=PSMD3 PE=1 SV=2	8	7	1.14
Q14681	BTB/POZ domain-containing protein KCTD2 OS=Homo sapiens OX=9606 GN=KCTD2 PE=1 SV=3	10	9	1.11
Q13263	Transcription intermediary factor 1-beta OS=Homo sapiens OX=9606 GN=TRIM28 PE=1 SV=5	11	10	1.10
O14744	Protein arginine N-methyltransferase 5 OS=Homo sapiens OX=9606 GN=PRMT5 PE=1 SV=4	56	51	1.10
O60825	6-phosphofructo-2-kinase/fructose-2,6-bisphosphatase 2 OS=Homo sapiens OX=9606 GN=PFKFB2 PE=1 SV=2	14	13	1.08
P07900	Heat shock protein HSP 90-alpha OS=Homo sapiens OX=9606 GN=HSP90AA1 PE=1 SV=5	14	13	1.08
Q16875	6-phosphofructo-2-kinase/fructose-2,6-bisphosphatase 3 OS=Homo sapiens OX=9606 GN=PFKFB3 PE=1 SV=1	15	15	1.00
Q8WWY3	U4/U6 small nuclear ribonucleoprotein Prp31 OS=Homo sapiens OX=9606 GN=PRPF31 PE=1 SV=2	7	7	1.00
P49411	Elongation factor Tu, mitochondrial OS=Homo sapiens OX=9606 GN=TUFM PE=1 SV=2	6	6	1.00
Q15057	Arf-GAP with coiled-coil, ANK repeat and PH domain-containing protein 2 OS=Homo sapiens OX=9606 GN=ACAP2 PE=1 SV=3	6	6	1.00
Q09028	Histone-binding protein RBBP4 OS=Homo sapiens OX=9606 GN=RBBP4 PE=1 SV=3	4	4	1.00
O43318	Mitogen-activated protein kinase kinase kinase 7 OS=Homo sapiens OX=9606 GN=MAP3K7 PE=1 SV=1	4	4	1.00
P35580	Myosin-10 OS=Homo sapiens OX=9606 GN=MYH10 PE=1 SV=3	4	4	1.00
Q13200	26S proteasome non-ATPase regulatory subunit 2 OS=Homo sapiens OX=9606 GN=PSMD2 PE=1 SV=3	4	4	1.00
P62195	26S proteasome regulatory subunit 8 OS=Homo sapiens OX=9606 GN=PSMC5 PE=1 SV=1	3	3	1.00
P14923	Junction plakoglobin OS=Homo sapiens OX=9606 GN=JUP PE=1 SV=3	3	3	1.00
Q96AV8	Transcription factor E2F7 OS=Homo sapiens OX=9606 GN=E2F7 PE=1 SV=3	3	3	1.00
Q9Y265	RuvB-like 1 OS=Homo sapiens OX=9606 GN=RUVBL1 PE=1 SV=1	3	3	1.00
Q03252	Lamin-B2 OS=Homo sapiens OX=9606 GN=LMNB2 PE=1 SV=4	2	2	1.00
P28074	Proteasome subunit beta type-5 OS=Homo sapiens OX=9606 GN=PSMB5 PE=1 SV=3	2	2	1.00
Q9P035	Very-long-chain (3R)-3-hydroxyacyl-CoA dehydratase 3 OS=Homo sapiens OX=9606 GN=HACD3 PE=1 SV=2	2	2	1.00
Q13162	Peroxiredoxin-4 OS=Homo sapiens OX=9606 GN=PRDX4 PE=1 SV=1	2	2	1.00
Q06830	Peroxiredoxin-1 OS=Homo sapiens OX=9606 GN=PRDX1 PE=1 SV=1	2	2	1.00
O60814	Histone H2B type 1-K OS=Homo sapiens OX=9606 GN=HIST1H2BK PE=1 SV=3	2	2	1.00
Q92922	SWI/SNF complex subunit SMARCC1 OS=Homo sapiens OX=9606 GN=SMARCC1 PE=1 SV=3	2	2	1.00
P30041	Peroxiredoxin-6 OS=Homo sapiens OX=9606 GN=PRDX6 PE=1 SV=3	2	2	1.00
P62333	26S proteasome regulatory subunit 10B OS=Homo sapiens OX=9606 GN=PSMC6 PE=1 SV=1	2	2	1.00
P55036	26S proteasome non-ATPase regulatory subunit 4 OS=Homo sapiens OX=9606 GN=PSMD4 PE=1 SV=1	2	2	1.00
Q15517	Corneodesmosin OS=Homo sapiens OX=9606 GN=CDSN PE=1 SV=3	2	2	1.00
Q8TDI0	Chromodomain-helicase-DNA-binding protein 5 OS=Homo sapiens OX=9606 GN=CHD5 PE=1 SV=1	1	1	1.00
Q00325	Phosphate carrier protein, mitochondrial OS=Homo sapiens OX=9606 GN=SLC25A3 PE=1 SV=2	1	1	1.00
P55084	Trifunctional enzyme subunit beta, mitochondrial OS=Homo sapiens OX=9606 GN=HADHB PE=1 SV=3	1	1	1.00
Q13428	Treacle protein OS=Homo sapiens OX=9606 GN=TCOF1 PE=1 SV=3	1	1	1.00
P06748	Nucleophosmin OS=Homo sapiens OX=9606 GN=NPM1 PE=1 SV=2	1	1	1.00
Q15459	Splicing factor 3A subunit 1 OS=Homo sapiens OX=9606 GN=SF3A1 PE=1 SV=1	1	1	1.00
P12268	Inosine-5'-monophosphate dehydrogenase 2 OS=Homo sapiens OX=9606 GN=IMPDH2 PE=1 SV=2	1	1	1.00
O00487	26S proteasome non-ATPase regulatory subunit 14 OS=Homo sapiens OX=9606 GN=PSMD14 PE=1 SV=1	1	1	1.00
Q3V6T2	Girdin OS=Homo sapiens OX=9606 GN=CCDC88A PE=1 SV=2	1	1	1.00
P25786	Proteasome subunit alpha type-1 OS=Homo sapiens OX=9606 GN=PSMA1 PE=1 SV=1	1	1	1.00
P28072	Proteasome subunit beta type-6 OS=Homo sapiens OX=9606 GN=PSMB6 PE=1 SV=4	1	1	1.00
P57088	Transmembrane protein 33 OS=Homo sapiens OX=9606 GN=TMEM33 PE=1 SV=2	1	1	1.00
Q9Y4Z0	U6 snRNA-associated Sm-like protein LSm4 OS=Homo sapiens OX=9606 GN=LSM4 PE=1 SV=1	1	1	1.00
O60763	General vesicular transport factor p115 OS=Homo sapiens OX=9606 GN=USO1 PE=1 SV=2	1	1	1.00
P28288	ATP-binding cassette sub-family D member 3 OS=Homo sapiens OX=9606 GN=ABCD3 PE=1 SV=1	1	1	1.00
Q99567	Nuclear pore complex protein Nup88 OS=Homo sapiens OX=9606 GN=NUP88 PE=1 SV=2	1	1	1.00
Q03393	6-pyruvoyl tetrahydrobiopterin synthase OS=Homo sapiens OX=9606 GN=PTS PE=1 SV=1	1	1	1.00
Q15758	Neutral amino acid transporter B(0) OS=Homo sapiens OX=9606 GN=SLC1A5 PE=1 SV=2	1	1	1.00
O75694	Nuclear pore complex protein Nup155 OS=Homo sapiens OX=9606 GN=NUP155 PE=1 SV=1	1	1	1.00
O15372	Eukaryotic translation initiation factor 3 subunit H OS=Homo sapiens OX=9606 GN=EIF3H PE=1 SV=1	1	1	1.00
P62937	Peptidyl-prolyl cis-trans isomerase A OS=Homo sapiens OX=9606 GN=PPIA PE=1 SV=2	1	1	1.00
Q9NYF8	Bcl-2-associated transcription factor 1 OS=Homo sapiens OX=9606 GN=BCLAF1 PE=1 SV=2	1	1	1.00
O75821	Eukaryotic translation initiation factor 3 subunit G OS=Homo sapiens OX=9606 GN=EIF3G PE=1 SV=2	1	1	1.00
Q9NVA2	Septin-11 OS=Homo sapiens OX=9606 GN=SEPT11 PE=1 SV=3	1	1	1.00
P13639	Elongation factor 2 OS=Homo sapiens OX=9606 GN=EEF2 PE=1 SV=4	1	1	1.00
P61221	ATP-binding cassette sub-family E member 1 OS=Homo sapiens OX=9606 GN=ABCE1 PE=1 SV=1	1	1	1.00
Q01804	OTU domain-containing protein 4 OS=Homo sapiens OX=9606 GN=OTUD4 PE=1 SV=4	1	1	1.00
Q6UUV7	CREB-regulated transcription coactivator 3 OS=Homo sapiens OX=9606 GN=CRTC3 PE=1 SV=2	1	1	1.00
Q8NC51	Plasminogen activator inhibitor 1 RNA-binding protein OS=Homo sapiens OX=9606 GN=SERBP1 PE=1 SV=2	1	1	1.00
P31153	S-adenosylmethionine synthase isoform type-2 OS=Homo sapiens OX=9606 GN=MAT2A PE=1 SV=1	1	1	1.00
P46060	Ran GTPase-activating protein 1 OS=Homo sapiens OX=9606 GN=RANGAP1 PE=1 SV=1	1	1	1.00
P23528	Cofilin-1 OS=Homo sapiens OX=9606 GN=CFL1 PE=1 SV=3	1	1	1.00
O75688	Protein phosphatase 1B OS=Homo sapiens OX=9606 GN=PPM1B PE=1 SV=1	69	71	0.97
P02768	Serum albumin OS=Homo sapiens OX=9606 GN=ALB PE=1 SV=2	18	20	0.90
Q8N5Z5	BTB/POZ domain-containing protein KCTD17 OS=Homo sapiens OX=9606 GN=KCTD17 PE=1 SV=3	6	7	0.86
P52732	Kinesin-like protein KIF11 OS=Homo sapiens OX=9606 GN=KIF11 PE=1 SV=2	16	19	0.84
Q14152	Eukaryotic translation initiation factor 3 subunit A OS=Homo sapiens OX=9606 GN=EIF3A PE=1 SV=1	9	11	0.82
Q07021	Complement component 1 Q subcomponent-binding protein, mitochondrial OS=Homo sapiens OX=9606 GN=C1QBP PE=1 SV=1	4	5	0.80
P62191	26S proteasome regulatory subunit 4 OS=Homo sapiens OX=9606 GN=PSMC1 PE=1 SV=1	4	5	0.80
P29692	Elongation factor 1-delta OS=Homo sapiens OX=9606 GN=EEF1D PE=1 SV=5	7	9	0.78
P98175	RNA-binding protein 10 OS=Homo sapiens OX=9606 GN=RBM10 PE=1 SV=3	38	50	0.76
Q8N1N4	Keratin, type II cytoskeletal 78 OS=Homo sapiens OX=9606 GN=KRT78 PE=1 SV=2	3	4	0.75
P08670	Vimentin OS=Homo sapiens OX=9606 GN=VIM PE=1 SV=4	5	7	0.71
Q9BQA1	Methylosome protein 50 OS=Homo sapiens OX=9606 GN=WDR77 PE=1 SV=1	16	23	0.70
P24534	Elongation factor 1-beta OS=Homo sapiens OX=9606 GN=EEF1B2 PE=1 SV=3	2	3	0.67
P05089	Arginase-1 OS=Homo sapiens OX=9606 GN=ARG1 PE=1 SV=2	2	3	0.67
Q8N5C8	TGF-beta-activated kinase 1 and MAP3K7-binding protein 3 OS=Homo sapiens OX=9606 GN=TAB3 PE=1 SV=3	2	3	0.67
Q01844	RNA-binding protein EWS OS=Homo sapiens OX=9606 GN=EWSR1 PE=1 SV=1	2	3	0.67
Q16576	Histone-binding protein RBBP7 OS=Homo sapiens OX=9606 GN=RBBP7 PE=1 SV=1	2	3	0.67
Q7L2H7	Eukaryotic translation initiation factor 3 subunit M OS=Homo sapiens OX=9606 GN=EIF3M PE=1 SV=1	2	3	0.67
Q8IWI9	MAX gene-associated protein OS=Homo sapiens OX=9606 GN=MGA PE=1 SV=3	2	3	0.67
Q7Z794	Keratin, type II cytoskeletal 1b OS=Homo sapiens OX=9606 GN=KRT77 PE=2 SV=3	12	19	0.63
P31943	Heterogeneous nuclear ribonucleoprotein H OS=Homo sapiens OX=9606 GN=HNRNPH1 PE=1 SV=4	5	8	0.63
P60709	Actin, cytoplasmic 1 OS=Homo sapiens OX=9606 GN=ACTB PE=1 SV=1	9	15	0.60
P26641	Elongation factor 1-gamma OS=Homo sapiens OX=9606 GN=EEF1G PE=1 SV=3	6	10	0.60
P35579	Myosin-9 OS=Homo sapiens OX=9606 GN=MYH9 PE=1 SV=4	3	5	0.60
P06576	ATP synthase subunit beta, mitochondrial OS=Homo sapiens OX=9606 GN=ATP5F1B PE=1 SV=3	13	23	0.57
Q9BUA3	SPIN1-docking protein OS=Homo sapiens OX=9606 GN=C11orf84 PE=1 SV=3	11	20	0.55
P13647	Keratin, type II cytoskeletal 5 OS=Homo sapiens OX=9606 GN=KRT5 PE=1 SV=3	17	31	0.55
P78347	General transcription factor II-I OS=Homo sapiens OX=9606 GN=GTF2I PE=1 SV=2	5	10	0.50
Q15019	Septin-2 OS=Homo sapiens OX=9606 GN=SEPT2 PE=1 SV=1	3	6	0.50
P17980	26S proteasome regulatory subunit 6A OS=Homo sapiens OX=9606 GN=PSMC3 PE=1 SV=3	2	4	0.50
P81605	Dermcidin OS=Homo sapiens OX=9606 GN=DCD PE=1 SV=2	1	2	0.50
P78332	RNA-binding protein 6 OS=Homo sapiens OX=9606 GN=RBM6 PE=1 SV=5	1	2	0.50
Q86YZ3	Hornerin OS=Homo sapiens OX=9606 GN=HRNR PE=1 SV=2	1	2	0.50
P51665	26S proteasome non-ATPase regulatory subunit 7 OS=Homo sapiens OX=9606 GN=PSMD7 PE=1 SV=2	1	2	0.50
Q12996	Cleavage stimulation factor subunit 3 OS=Homo sapiens OX=9606 GN=CSTF3 PE=1 SV=1	1	2	0.50
Q14203	Dynactin subunit 1 OS=Homo sapiens OX=9606 GN=DCTN1 PE=1 SV=3	1	2	0.50
Q13347	Eukaryotic translation initiation factor 3 subunit I OS=Homo sapiens OX=9606 GN=EIF3I PE=1 SV=1	1	2	0.50
P22314	Ubiquitin-like modifier-activating enzyme 1 OS=Homo sapiens OX=9606 GN=UBA1 PE=1 SV=3	1	2	0.50
P25788	Proteasome subunit alpha type-3 OS=Homo sapiens OX=9606 GN=PSMA3 PE=1 SV=2	1	2	0.50
P15924	Desmoplakin OS=Homo sapiens OX=9606 GN=DSP PE=1 SV=3	9	20	0.45
Q9BRS2	Serine/threonine-protein kinase RIO1 OS=Homo sapiens OX=9606 GN=RIOK1 PE=1 SV=2	2	5	0.40
P04406	Glyceraldehyde-3-phosphate dehydrogenase OS=Homo sapiens OX=9606 GN=GAPDH PE=1 SV=3	2	5	0.40
P02533	Keratin, type I cytoskeletal 14 OS=Homo sapiens OX=9606 GN=KRT14 PE=1 SV=4	9	25	0.36
O00303	Eukaryotic translation initiation factor 3 subunit F OS=Homo sapiens OX=9606 GN=EIF3F PE=1 SV=1	1	3	0.33
P19338	Nucleolin OS=Homo sapiens OX=9606 GN=NCL PE=1 SV=3	1	3	0.33
Q9Y2W1	Thyroid hormone receptor-associated protein 3 OS=Homo sapiens OX=9606 GN=THRAP3 PE=1 SV=2	1	3	0.33
Q02413	Desmoglein-1 OS=Homo sapiens OX=9606 GN=DSG1 PE=1 SV=2	3	10	0.30
P35527	Keratin, type I cytoskeletal 9 OS=Homo sapiens OX=9606 GN=KRT9 PE=1 SV=3	20	79	0.25
Q9NVH6	Trimethyllysine dioxygenase, mitochondrial OS=Homo sapiens OX=9606 GN=TMLHE PE=1 SV=1	1	4	0.25
P62805	Histone H4 OS=Homo sapiens OX=9606 GN=HIST1H4A PE=1 SV=2	1	4	0.25
P49366	Deoxyhypusine synthase OS=Homo sapiens OX=9606 GN=DHPS PE=1 SV=1	1	5	0.20
Q00839	Heterogeneous nuclear ribonucleoprotein U OS=Homo sapiens OX=9606 GN=HNRNPU PE=1 SV=6	1	5	0.20
Q92973	Transportin-1 OS=Homo sapiens OX=9606 GN=TNPO1 PE=1 SV=2	14		
B5ME19	Eukaryotic translation initiation factor 3 subunit C-like protein OS=Homo sapiens OX=9606 GN=EIF3CL PE=3 SV=1	11		

P02538	Keratin, type II cytoskeletal 6A OS=Homo sapiens OX=9606 GN=KRT6A PE=1 SV=3	10		
Q92621	Nuclear pore complex protein Nup205 OS=Homo sapiens OX=9606 GN=NUP205 PE=1 SV=3	10		
P12236	ADP/ATP translocase 3 OS=Homo sapiens OX=9606 GN=SLC25A6 PE=1 SV=4	10		
P49792	E3 SUMO-protein ligase RanBP2 OS=Homo sapiens OX=9606 GN=RANBP2 PE=1 SV=2	8		
Q9NRY5	Protein FAM114A2 OS=Homo sapiens OX=9606 GN=FAM114A2 PE=1 SV=4	8		
O43592	Exportin-T OS=Homo sapiens OX=9606 GN=XPOT PE=1 SV=2	7		
P08195	4F2 cell-surface antigen heavy chain OS=Homo sapiens OX=9606 GN=SLC3A2 PE=1 SV=3	7		
Q9C0E2	Exportin-4 OS=Homo sapiens OX=9606 GN=XPO4 PE=1 SV=2	6		
Q8IVL1	Neuron navigator 2 OS=Homo sapiens OX=9606 GN=NAV2 PE=1 SV=3	6		
P27348	14-3-3 protein theta OS=Homo sapiens OX=9606 GN=YWHAQ PE=1 SV=1	6		
O75150	E3 ubiquitin-protein ligase BRE1B OS=Homo sapiens OX=9606 GN=RNF40 PE=1 SV=4	6		
Q9H583	HEAT repeat-containing protein 1 OS=Homo sapiens OX=9606 GN=HEATR1 PE=1 SV=3	6		
P41252	Isoleucine--tRNA ligase, cytoplasmic OS=Homo sapiens OX=9606 GN=IARS PE=1 SV=2	5		
P27824	Calnexin OS=Homo sapiens OX=9606 GN=CANX PE=1 SV=2	5		
Q86Y56	Dynein assembly factor 5, axonemal OS=Homo sapiens OX=9606 GN=DNAAF5 PE=1 SV=4	5		
Q9Y5M8	Signal recognition particle receptor subunit beta OS=Homo sapiens OX=9606 GN=SRPRB PE=1 SV=3	5		
P55786	Puromycin-sensitive aminopeptidase OS=Homo sapiens OX=9606 GN=NPEPPS PE=1 SV=2	5		
Q93008	Probable ubiquitin carboxyl-terminal hydrolase FAF-X OS=Homo sapiens OX=9606 GN=USP9X PE=1 SV=3	5		
Q9NQ92	Coordinator of PRMT5 and differentiation stimulator OS=Homo sapiens OX=9606 GN=COPRS PE=1 SV=3	5		
P35658	Nuclear pore complex protein Nup214 OS=Homo sapiens OX=9606 GN=NUP214 PE=1 SV=2	5		
P34932	Heat shock 70 kDa protein 4 OS=Homo sapiens OX=9606 GN=HSPA4 PE=1 SV=4	4		
P62736	Actin, aortic smooth muscle OS=Homo sapiens OX=9606 GN=ACTA2 PE=1 SV=1	4		
Q14CN4	Keratin, type II cytoskeletal 72 OS=Homo sapiens OX=9606 GN=KRT72 PE=1 SV=2	4		
Q8N3U4	Cohesin subunit SA-2 OS=Homo sapiens OX=9606 GN=STAG2 PE=1 SV=3	4		
Q9UBB4	Ataxin-10 OS=Homo sapiens OX=9606 GN=ATXN10 PE=1 SV=1	4		
O15260	Surfeit locus protein 4 OS=Homo sapiens OX=9606 GN=SURF4 PE=1 SV=3	4		
Q5VTR2	E3 ubiquitin-protein ligase BRE1A OS=Homo sapiens OX=9606 GN=RNF20 PE=1 SV=2	4		
P56192	Methionine--tRNA ligase, cytoplasmic OS=Homo sapiens OX=9606 GN=MARS PE=1 SV=2	4		
O00232	26S proteasome non-ATPase regulatory subunit 12 OS=Homo sapiens OX=9606 GN=PSMD12 PE=1 SV=3	4		
P49321	Nuclear autoantigenic sperm protein OS=Homo sapiens OX=9606 GN=NASP PE=1 SV=2	4		
P20020	Plasma membrane calcium-transporting ATPase 1 OS=Homo sapiens OX=9606 GN=ATP2B1 PE=1 SV=3	3		
Q15021	Condensin complex subunit 1 OS=Homo sapiens OX=9606 GN=NCAPD2 PE=1 SV=3	3		
Q9Y6Y0	Influenza virus NS1A-binding protein OS=Homo sapiens OX=9606 GN=IVNS1ABP PE=1 SV=3	3		
Q5JPE7	Nodal modulator 2 OS=Homo sapiens OX=9606 GN=NOMO2 PE=1 SV=1	3		
P12277	Creatine kinase B-type OS=Homo sapiens OX=9606 GN=CKB PE=1 SV=1	3		
O94906	Pre-mRNA-processing factor 6 OS=Homo sapiens OX=9606 GN=PRPF6 PE=1 SV=1	3		
P54136	Arginine--tRNA ligase, cytoplasmic OS=Homo sapiens OX=9606 GN=RARS PE=1 SV=2	3		
P40939	Trifunctional enzyme subunit alpha, mitochondrial OS=Homo sapiens OX=9606 GN=HADHA PE=1 SV=2	3		
P42167	Lamina-associated polypeptide 2, isoforms beta/gamma OS=Homo sapiens OX=9606 GN=TMPO PE=1 SV=2	3		
Q3LXA3	Triokinase/FMN cyclase OS=Homo sapiens OX=9606 GN=TKFC PE=1 SV=2	3		
Q14315	Filamin-C OS=Homo sapiens OX=9606 GN=FLNC PE=1 SV=3	3		
O75146	Huntingtin-interacting protein 1-related protein OS=Homo sapiens OX=9606 GN=HIP1R PE=1 SV=2	3		
Q16181	Septin-7 OS=Homo sapiens OX=9606 GN=SEPT7 PE=1 SV=2	3		
Q14683	Structural maintenance of chromosomes protein 1A OS=Homo sapiens OX=9606 GN=SMC1A PE=1 SV=2	3		
P31689	DnaJ homolog subfamily A member 1 OS=Homo sapiens OX=9606 GN=DNAJA1 PE=1 SV=2	3		
Q14697	Neutral alpha-glucosidase AB OS=Homo sapiens OX=9606 GN=GANAB PE=1 SV=3	3		
Q9HAV4	Exportin-5 OS=Homo sapiens OX=9606 GN=XPO5 PE=1 SV=1	3		
Q9P2E9	Ribosome-binding protein 1 OS=Homo sapiens OX=9606 GN=RRBP1 PE=1 SV=5	3		
P48740	Mannan-binding lectin serine protease 1 OS=Homo sapiens OX=9606 GN=MASP1 PE=1 SV=3	2		
P36542	ATP synthase subunit gamma, mitochondrial OS=Homo sapiens OX=9606 GN=ATP5F1C PE=1 SV=1	2		
P46977	Dolichyl-diphosphooligosaccharide --protein glycosyltransferase subunit STT3A OS=Homo sapiens OX=9606 GN=STT3A PE=1 SV=2	2		
Q7Z4Q2	HEAT repeat-containing protein 3 OS=Homo sapiens OX=9606 GN=HEATR3 PE=1 SV=2	2		
O00461	Golgi integral membrane protein 4 OS=Homo sapiens OX=9606 GN=GOLIM4 PE=1 SV=1	2		
Q8TEX9	Importin-4 OS=Homo sapiens OX=9606 GN=IPO4 PE=1 SV=2	2		
P42357	Histidine ammonia-lyase OS=Homo sapiens OX=9606 GN=HAL PE=1 SV=1	2		
Q9Y6D9	Mitotic spindle assembly checkpoint protein MAD1 OS=Homo sapiens OX=9606 GN=MAD1L1 PE=1 SV=2	2		
O14654	Insulin receptor substrate 4 OS=Homo sapiens OX=9606 GN=IRS4 PE=1 SV=1	2		
P49790	Nuclear pore complex protein Nup153 OS=Homo sapiens OX=9606 GN=NUP153 PE=1 SV=2	2		
Q9H3U1	Protein unc-45 homolog A OS=Homo sapiens OX=9606 GN=UNC45A PE=1 SV=1	2		
Q9Y277	Voltage-dependent anion-selective channel protein 3 OS=Homo sapiens OX=9606 GN=VDAC3 PE=1 SV=1	2		
P50851	Lipopolysaccharide -responsive and beige-like anchor protein OS=Homo sapiens OX=9606 GN=LRBA PE=1 SV=4	2		
P51610	Host cell factor 1 OS=Homo sapiens OX=9606 GN=HCFC1 PE=1 SV=2	2		
Q86TV6	Tetratricopeptide repeat protein 7B OS=Homo sapiens OX=9606 GN=TTC7B PE=1 SV=3	2		
Q96P70	Importin-9 OS=Homo sapiens OX=9606 GN=IPO9 PE=1 SV=3	2		
Q8TC07	TBC1 domain family member 15 OS=Homo sapiens OX=9606 GN=TBC1D15 PE=1 SV=2	2		
O43175	D-3-phosphoglycerate dehydrogenase OS=Homo sapiens OX=9606 GN=PHGDH PE=1 SV=4	2		
P20618	Proteasome subunit beta type-1 OS=Homo sapiens OX=9606 GN=PSMB1 PE=1 SV=2	2		
Q9BXW9	Fanconi anemia group D2 protein OS=Homo sapiens OX=9606 GN=FANCD2 PE=1 SV=2	2		
Q70IA6	MOB kinase activator 2 OS=Homo sapiens OX=9606 GN=MOB2 PE=1 SV=1	2		
Q9BZX2	Uridine-cytidine kinase 2 OS=Homo sapiens OX=9606 GN=UCK2 PE=1 SV=1	2		
Q9Y2A7	Nck-associated protein 1 OS=Homo sapiens OX=9606 GN=NCKAP1 PE=1 SV=1	2		
Q5SRE5	Nucleoporin NUP188 homolog OS=Homo sapiens OX=9606 GN=NUP188 PE=1 SV=1	2		
P30613	Pyruvate kinase PKLR OS=Homo sapiens OX=9606 GN=PKLR PE=1 SV=2	2		
O60610	Protein diaphanous homolog 1 OS=Homo sapiens OX=9606 GN=DIAPH1 PE=1 SV=2	2		
P05387	60S acidic ribosomal protein P2 OS=Homo sapiens OX=9606 GN=RPLP2 PE=1 SV=1	2		
Q6UB35	Monofunctional C1-tetrahydrofolate synthase, mitochondrial OS=Homo sapiens OX=9606 GN=MTHFD1L PE=1 SV=1	2		
Q9UPQ9	Trinucleotide repeat-containing gene 6B protein OS=Homo sapiens OX=9606 GN=TNRC6B PE=1 SV=4	2		
Q5UIP0	Telomere-associated protein RIF1 OS=Homo sapiens OX=9606 GN=RIF1 PE=1 SV=2	2		
P45880	Voltage-dependent anion-selective channel protein 2 OS=Homo sapiens OX=9606 GN=VDAC2 PE=1 SV=2	2		
Q9Y5Q9	General transcription factor 3C polypeptide 3 OS=Homo sapiens OX=9606 GN=GTF3C3 PE=1 SV=1	1		
Q9UBV2	Protein sel-1 homolog 1 OS=Homo sapiens OX=9606 GN=SEL1L PE=1 SV=3	1		
Q9UPU5	Ubiquitin carboxyl-terminal hydrolase 24 OS=Homo sapiens OX=9606 GN=USP24 PE=1 SV=3	1		
Q8NCE2	Myotubularin-related protein 14 OS=Homo sapiens OX=9606 GN=MTMR14 PE=1 SV=2	1		
Q14240	Eukaryotic initiation factor 4A-II OS=Homo sapiens OX=9606 GN=EIF4A2 PE=1 SV=2	1		
O95239	Chromosome-associated kinesin KIF4A OS=Homo sapiens OX=9606 GN=KIF4A PE=1 SV=3	1		
O75607	Nucleoplasmin-3 OS=Homo sapiens OX=9606 GN=NPM3 PE=1 SV=3	1		
Q96ER3	Protein SAAL1 OS=Homo sapiens OX=9606 GN=SAAL1 PE=1 SV=2	1		
Q9UFF9	CCR4-NOT transcription complex subunit 8 OS=Homo sapiens OX=9606 GN=CNOT8 PE=1 SV=1	1		
Q96JS3	PiggyBac transposable element-derived protein 1 OS=Homo sapiens OX=9606 GN=PGBD1 PE=1 SV=1	1		
P41250	Glycine--tRNA ligase OS=Homo sapiens OX=9606 GN=GARS PE=1 SV=3	1		
Q7L1Q6	Basic leucine zipper and W2 domain-containing protein 1 OS=Homo sapiens OX=9606 GN=BZW1 PE=1 SV=1	1		
Q9NU22	Midasin OS=Homo sapiens OX=9606 GN=MDN1 PE=1 SV=2	1		
P46940	Ras GTPase-activating-like protein IQGAP1 OS=Homo sapiens OX=9606 GN=IQGAP1 PE=1 SV=1	1		
Q86UP2	Kinectin OS=Homo sapiens OX=9606 GN=KTN1 PE=1 SV=1	1		
P12004	Proliferating cell nuclear antigen OS=Homo sapiens OX=9606 GN=PCNA PE=1 SV=1	1		
Q96KP1	Exocyst complex component 2 OS=Homo sapiens OX=9606 GN=EXOC2 PE=1 SV=1	1		
O00767	Acyl-CoA desaturase OS=Homo sapiens OX=9606 GN=SCD PE=1 SV=2	1		
Q9Y5K6	CD2-associated protein OS=Homo sapiens OX=9606 GN=CD2AP PE=1 SV=1	1		
P09622	Dihydrolipoyl dehydrogenase, mitochondrial OS=Homo sapiens OX=9606 GN=DLD PE=1 SV=2	1		
Q9NZ01	Very-long-chain enoyl-CoA reductase OS=Homo sapiens OX=9606 GN=TECR PE=1 SV=1	1		
Q969Z0	FAST kinase domain-containing protein 4 OS=Homo sapiens OX=9606 GN=TBRG4 PE=1 SV=1	1		
P08758	Annexin A5 OS=Homo sapiens OX=9606 GN=ANXA5 PE=1 SV=2	1		
Q08554	Desmocollin-1 OS=Homo sapiens OX=9606 GN=DSC1 PE=1 SV=2	1		
O95983	Methyl-CpG-binding domain protein 3 OS=Homo sapiens OX=9606 GN=MBD3 PE=1 SV=1	1		
Q96QU8	Exportin-6 OS=Homo sapiens OX=9606 GN=XPO6 PE=1 SV=1	1		
Q8NF91	Nesprin-1 OS=Homo sapiens OX=9606 GN=SYNE1 PE=1 SV=4	1		
Q8IUD2	ELKS/Rab6-interacting/CAST family member 1 OS=Homo sapiens OX=9606 GN=ERC1 PE=1 SV=1	1		
O95394	Phosphoacetylglucosamine mutase OS=Homo sapiens OX=9606 GN=PGM3 PE=1 SV=1	1		
P19022	Cadherin-2 OS=Homo sapiens OX=9606 GN=CDH2 PE=1 SV=4	1		
Q9Y6E2	Basic leucine zipper and W2 domain-containing protein 2 OS=Homo sapiens OX=9606 GN=BZW2 PE=1 SV=1	1		
P35221	Catenin alpha-1 OS=Homo sapiens OX=9606 GN=CTNNA1 PE=1 SV=1	1		
Q9UMZ2	Synergin gamma OS=Homo sapiens OX=9606 GN=SYNRG PE=1 SV=2	1		
P42566	Epidermal growth factor receptor substrate 15 OS=Homo sapiens OX=9606 GN=EPS15 PE=1 SV=2	1		
Q9Y4W6	AFG3-like protein 2 OS=Homo sapiens OX=9606 GN=AFG3L2 PE=1 SV=2	1		
Q9Y5L0	Transportin-3 OS=Homo sapiens OX=9606 GN=TNPO3 PE=1 SV=3	1		
Q8IUH8	Signal peptide peptidase-like 2C OS=Homo sapiens OX=9606 GN=SPPL2C PE=1 SV=3	1		
Q5JRA6	Transport and Golgi organization protein 1 homolog OS=Homo sapiens OX=9606 GN=MIA3 PE=1 SV=1	1		
Q99956	Dual specificity protein phosphatase 9 OS=Homo sapiens OX=9606 GN=DUSP9 PE=1 SV=1	1		
O75533	Splicing factor 3B subunit 1 OS=Homo sapiens OX=9606 GN=SF3B1 PE=1 SV=3	1		
Q15149	Plectin OS=Homo sapiens OX=9606 GN=PLEC PE=1 SV=3	1		
P25787	Proteasome subunit alpha type-2 OS=Homo sapiens OX=9606 GN=PSMA2 PE=1 SV=2	1		
Q8TD84	Down syndrome cell adhesion molecule-like protein 1 OS=Homo sapiens OX=9606 GN=DSCAML1 PE=1 SV=2	1		
Q9BSJ2	Gamma-tubulin complex component 2 OS=Homo sapiens OX=9606 GN=TUBGCP2 PE=1 SV=2	1		
Q9NVI1	Fanconi anemia group I protein OS=Homo sapiens OX=9606 GN=FANCI PE=1 SV=4	1		
O43166	Signal-induced proliferation-associated 1-like protein 1 OS=Homo sapiens OX=9606 GN=SIPA1L1 PE=1 SV=4	1		
P48556	26S proteasome non-ATPase regulatory subunit 8 OS=Homo sapiens OX=9606 GN=PSMD8 PE=1 SV=2	1		
Q9P003	Protein cornichon homolog 4 OS=Homo sapiens OX=9606 GN=CNIH4 PE=1 SV=1	1		
P30050	60S ribosomal protein L12 OS=Homo sapiens OX=9606 GN=RPL12 PE=1 SV=1	1		
Q9BT22	Chitobiosyldiphosphodolichol beta-mannosyltransferase OS=Homo sapiens OX=9606 GN=ALG1 PE=1 SV=2	1		
Q2M389	WASH complex subunit 4 OS=Homo sapiens OX=9606 GN=WASHC4 PE=1 SV=2	1		
Q9NRZ9	Lymphoid-specific helicase OS=Homo sapiens OX=9606 GN=HELLS PE=1 SV=1	1		
Q7Z353	Highly divergent homeobox OS=Homo sapiens OX=9606 GN=HDX PE=1 SV=1	1		

P61204	ADP-ribosylation factor 3 OS=Homo sapiens OX=9606 GN=ARF3 PE=1 SV=2	1		
O60313	Dynamin-like 120 kDa protein, mitochondrial OS=Homo sapiens OX=9606 GN=OPA1 PE=1 SV=3	1		
Q9UBC2	Epidermal growth factor receptor substrate 15-like 1 OS=Homo sapiens OX=9606 GN=EPS15L1 PE=1 SV=1	1		
Q8WUM0	Nuclear pore complex protein Nup133 OS=Homo sapiens OX=9606 GN=NUP133 PE=1 SV=2	1		
Q9NQX3	Gephyrin OS=Homo sapiens OX=9606 GN=GPHN PE=1 SV=1	1		
Q9Y263	Phospholipase A-2-activating protein OS=Homo sapiens OX=9606 GN=PLAA PE=1 SV=2	1		
Q9UPT5	Exocyst complex component 7 OS=Homo sapiens OX=9606 GN=EXOC7 PE=1 SV=3	1		
P28066	Proteasome subunit alpha type-5 OS=Homo sapiens OX=9606 GN=PSMA5 PE=1 SV=3	1		
Q53GQ0	Very-long-chain 3-oxoacyl-CoA reductase OS=Homo sapiens OX=9606 GN=HSD17B12 PE=1 SV=2	1		
O75197	Low-density lipoprotein receptor-related protein 5 OS=Homo sapiens OX=9606 GN=LRP5 PE=1 SV=2	1		
Q9Y4E8	Ubiquitin carboxyl-terminal hydrolase 15 OS=Homo sapiens OX=9606 GN=USP15 PE=1 SV=3	1		
Q8WVX9	Fatty acyl-CoA reductase 1 OS=Homo sapiens OX=9606 GN=FAR1 PE=1 SV=1	1		
O60216	Double-strand-break repair protein rad21 homolog OS=Homo sapiens OX=9606 GN=RAD21 PE=1 SV=2	1		
Q9Y6A5	Transforming acidic coiled-coil-containing protein 3 OS=Homo sapiens OX=9606 GN=TACC3 PE=1 SV=1	1		
Q5VYK3	Proteasome adapter and scaffold protein ECM29 OS=Homo sapiens OX=9606 GN=ECPAS PE=1 SV=2	1		
O60762	Dolichol-phosphate mannosyltransferase subunit 1 OS=Homo sapiens OX=9606 GN=DPM1 PE=1 SV=1	1		
P28070	Proteasome subunit beta type-4 OS=Homo sapiens OX=9606 GN=PSMB4 PE=1 SV=4	1		
P55735	Protein SEC13 homolog OS=Homo sapiens OX=9606 GN=SEC13 PE=1 SV=3	1		
P61626	Lysozyme C OS=Homo sapiens OX=9606 GN=LYZ PE=1 SV=1	1		
Q14004	Cyclin-dependent kinase 13 OS=Homo sapiens OX=9606 GN=CDK13 PE=1 SV=2	1		
P30622	CAP-Gly domain-containing linker protein 1 OS=Homo sapiens OX=9606 GN=CLIP1 PE=1 SV=2	1		
P48651	Phosphatidylserine synthase 1 OS=Homo sapiens OX=9606 GN=PTDSS1 PE=1 SV=1	1		
P50570	Dynamin-2 OS=Homo sapiens OX=9606 GN=DNM2 PE=1 SV=2	1		
P62913	60S ribosomal protein L11 OS=Homo sapiens OX=9606 GN=RPL11 PE=1 SV=2	1		
P10589	COUP transcription factor 1 OS=Homo sapiens OX=9606 GN=NR2F1 PE=1 SV=1	1		
O15294	UDP-N-acetylglucosamine--peptide N-acetylglucosaminyltransferase 110 kDa subunit OS=Homo sapiens OX=9606 GN=OGT PE=1 SV=3	1		
Q14204	Cytoplasmic dynein 1 heavy chain 1 OS=Homo sapiens OX=9606 GN=DYNC1H1 PE=1 SV=5	1		
Q13045	Protein flightless-1 homolog OS=Homo sapiens OX=9606 GN=FLII PE=1 SV=2	1		
Q8TCG1	Protein CIP2A OS=Homo sapiens OX=9606 GN=CIP2A PE=1 SV=2	1		
Q15276	Rab GTPase-binding effector protein 1 OS=Homo sapiens OX=9606 GN=RABEP1 PE=1 SV=2	1		
O43264	Centromere/kinetochore protein zw10 homolog OS=Homo sapiens OX=9606 GN=ZW10 PE=1 SV=3	1		
P31948	Stress-induced-phosphoprotein 1 OS=Homo sapiens OX=9606 GN=STIP1 PE=1 SV=1	1		
P51659	Peroxisomal multifunctional enzyme type 2 OS=Homo sapiens OX=9606 GN=HSD17B4 PE=1 SV=3	1		
O00255	Menin OS=Homo sapiens OX=9606 GN=MEN1 PE=1 SV=4	1		
Q03169	Tumor necrosis factor alpha-induced protein 2 OS=Homo sapiens OX=9606 GN=TNFAIP2 PE=2 SV=2	1		
Q32MZ4	Leucine-rich repeat flightless-interacting protein 1 OS=Homo sapiens OX=9606 GN=LRRFIP1 PE=1 SV=2	1		
P50748	Kinetochore-associated protein 1 OS=Homo sapiens OX=9606 GN=KNTC1 PE=1 SV=1	1		
Q92499	ATP-dependent RNA helicase DDX1 OS=Homo sapiens OX=9606 GN=DDX1 PE=1 SV=2	1		
Q9Y512	Sorting and assembly machinery component 50 homolog OS=Homo sapiens OX=9606 GN=SAMM50 PE=1 SV=3	1		
P53007	Tricarboxylate transport protein, mitochondrial OS=Homo sapiens OX=9606 GN=SLC25A1 PE=1 SV=2	1		
O95140	Mitofusin-2 OS=Homo sapiens OX=9606 GN=MFN2 PE=1 SV=3	1		
P01614	Immunoglobulin kappa variable 2D-40 OS=Homo sapiens OX=9606 GN=IGKV2D-40 PE=1 SV=2	1		
Q6KB66	Keratin, type II cytoskeletal 80 OS=Homo sapiens OX=9606 GN=KRT80 PE=1 SV=2		12	
P12035	Keratin, type II cytoskeletal 3 OS=Homo sapiens OX=9606 GN=KRT3 PE=1 SV=3		12	
Q3SY84	Keratin, type II cytoskeletal 71 OS=Homo sapiens OX=9606 GN=KRT71 PE=1 SV=3		10	
Q04695	Keratin, type I cytoskeletal 17 OS=Homo sapiens OX=9606 GN=KRT17 PE=1 SV=2		9	
P68871	Hemoglobin subunit beta OS=Homo sapiens OX=9606 GN=HBB PE=1 SV=2		8	
Q99613	Eukaryotic translation initiation factor 3 subunit C OS=Homo sapiens OX=9606 GN=EIF3C PE=1 SV=1		8	
A0A075B6S2	Immunoglobulin kappa variable 2D-29 OS=Homo sapiens OX=9606 GN=IGKV2D-29 PE=3 SV=1		7	
P55795	Heterogeneous nuclear ribonucleoprotein H2 OS=Homo sapiens OX=9606 GN=HNRNPH2 PE=1 SV=1		5	
P14618	Pyruvate kinase PKM OS=Homo sapiens OX=9606 GN=PKM PE=1 SV=4		4	
Q9UHX1	Poly(U)-binding-splicing factor PUF60 OS=Homo sapiens OX=9606 GN=PUF60 PE=1 SV=1		3	
P61163	Alpha-centractin OS=Homo sapiens OX=9606 GN=ACTR1A PE=1 SV=1		3	
P07355	Annexin A2 OS=Homo sapiens OX=9606 GN=ANXA2 PE=1 SV=2		3	
P04075	Fructose-bisphosphate aldolase A OS=Homo sapiens OX=9606 GN=ALDOA PE=1 SV=2		3	
P04908	Histone H2A type 1-B/E OS=Homo sapiens OX=9606 GN=HIST1H2AB PE=1 SV=2		3	
P22234	Multifunctional protein ADE2 OS=Homo sapiens OX=9606 GN=PAICS PE=1 SV=3		3	
P43243	Matrin-3 OS=Homo sapiens OX=9606 GN=MATR3 PE=1 SV=2		2	
P06858	Lipoprotein lipase OS=Homo sapiens OX=9606 GN=LPL PE=1 SV=1		2	
Q15233	Non-POU domain-containing octamer-binding protein OS=Homo sapiens OX=9606 GN=NONO PE=1 SV=4		2	
P12956	X-ray repair cross-complementing protein 6 OS=Homo sapiens OX=9606 GN=XRCC6 PE=1 SV=2		2	
P23246	Splicing factor, proline- and glutamine-rich OS=Homo sapiens OX=9606 GN=SFPQ PE=1 SV=2		2	
P07197	Neurofilament medium polypeptide OS=Homo sapiens OX=9606 GN=NEFM PE=1 SV=3		2	
P09874	Poly [ADP-ribose] polymerase 1 OS=Homo sapiens OX=9606 GN=PARP1 PE=1 SV=4		2	
P08865	40S ribosomal protein SA OS=Homo sapiens OX=9606 GN=RPSA PE=1 SV=4		2	
Q99873	Protein arginine N-methyltransferase 1 OS=Homo sapiens OX=9606 GN=PRMT1 PE=1 SV=3		2	
Q8ND56	Protein LSM14 homolog A OS=Homo sapiens OX=9606 GN=LSM14A PE=1 SV=3		1	
Q02978	Mitochondrial 2-oxoglutarate/malate carrier protein OS=Homo sapiens OX=9606 GN=SLC25A11 PE=1 SV=3		1	
P46783	40S ribosomal protein S10 OS=Homo sapiens OX=9606 GN=RPS10 PE=1 SV=1		1	
P23284	Peptidyl-prolyl cis-trans isomerase B OS=Homo sapiens OX=9606 GN=PPIB PE=1 SV=2		1	
P63151	Serine/threonine-protein phosphatase 2A 55 kDa regulatory subunit B alpha isoform OS=Homo sapiens OX=9606 GN=PPP2R2A PE=1 SV=1		1	
Q96HN2	Adenosylhomocysteinase 3 OS=Homo sapiens OX=9606 GN=AHCYL2 PE=1 SV=1		1	
Q15084	Protein disulfide-isomerase A6 OS=Homo sapiens OX=9606 GN=PDIA6 PE=1 SV=1		1	
O43390	Heterogeneous nuclear ribonucleoprotein R OS=Homo sapiens OX=9606 GN=HNRNPR PE=1 SV=1		1	
O75223	Gamma-glutamylcyclotransferase OS=Homo sapiens OX=9606 GN=GGCT PE=1 SV=1		1	
P18124	60S ribosomal protein L7 OS=Homo sapiens OX=9606 GN=RPL7 PE=1 SV=1		1	
P01857	Immunoglobulin heavy constant gamma 1 OS=Homo sapiens OX=9606 GN=IGHG1 PE=1 SV=1		1	
Q08188	Protein-glutamine gamma-glutamyltransferase E OS=Homo sapiens OX=9606 GN=TGM3 PE=1 SV=4		1	
O14974	Protein phosphatase 1 regulatory subunit 12A OS=Homo sapiens OX=9606 GN=PPP1R12A PE=1 SV=1		1	
Q8IVT5	Kinase suppressor of Ras 1 OS=Homo sapiens OX=9606 GN=KSR1 PE=1 SV=3		1	
P22735	Protein-glutamine gamma-glutamyltransferase K OS=Homo sapiens OX=9606 GN=TGM1 PE=1 SV=4		1	
P31025	Lipocalin-1 OS=Homo sapiens OX=9606 GN=LCN1 PE=1 SV=1		1	
Q14526	Hypermethylated in cancer 1 protein OS=Homo sapiens OX=9606 GN=HIC1 PE=1 SV=5		1	
Q9Y224	RNA transcription, translation and transport factor protein OS=Homo sapiens OX=9606 GN=RTRAF PE=1 SV=1		1	
P42166	Lamina-associated polypeptide 2, isoform alpha OS=Homo sapiens OX=9606 GN=TMPO PE=1 SV=2		1	
Q08211	ATP-dependent RNA helicase A OS=Homo sapiens OX=9606 GN=DHX9 PE=1 SV=4		1	
P69905	Hemoglobin subunit alpha OS=Homo sapiens OX=9606 GN=HBA1 PE=1 SV=2		1	
P49641	Alpha-mannosidase 2x OS=Homo sapiens OX=9606 GN=MAN2A2 PE=2 SV=3		1	
Q9NP73	Putative bifunctional UDP-N-acetylglucosamine transferase and deubiquitinase ALG13 OS=Homo sapiens OX=9606 GN=ALG13 PE=1 SV=2		1	
P29508	Serpin B3 OS=Homo sapiens OX=9606 GN=SERPINB3 PE=1 SV=2		1	
Q14160	Protein scribble homolog OS=Homo sapiens OX=9606 GN=SCRIB PE=1 SV=4		1	
Q8N1I0	Dedicator of cytokinesis protein 4 OS=Homo sapiens OX=9606 GN=DOCK4 PE=1 SV=3		1	
Q9BT78	COP9 signalosome complex subunit 4 OS=Homo sapiens OX=9606 GN=COPS4 PE=1 SV=1		1	
O14513	Nck-associated protein 5 OS=Homo sapiens OX=9606 GN=NCKAP5 PE=1 SV=2		1	
Q01518	Adenylyl cyclase-associated protein 1 OS=Homo sapiens OX=9606 GN=CAP1 PE=1 SV=5		1	
O00231	26S proteasome non-ATPase regulatory subunit 11 OS=Homo sapiens OX=9606 GN=PSMD11 PE=1 SV=3		1	
P28482	Mitogen-activated protein kinase 1 OS=Homo sapiens OX=9606 GN=MAPK1 PE=1 SV=3		1	
Q92841	Probable ATP-dependent RNA helicase DDX17 OS=Homo sapiens OX=9606 GN=DDX17 PE=1 SV=2		1	
Q9UBW8	COP9 signalosome complex subunit 7a OS=Homo sapiens OX=9606 GN=COPS7A PE=1 SV=1		1	
Q8IX12	Cell division cycle and apoptosis regulator protein 1 OS=Homo sapiens OX=9606 GN=CCAR1 PE=1 SV=2		1	
Q96P63	Serpin B12 OS=Homo sapiens OX=9606 GN=SERPINB12 PE=1 SV=1		1	
P22626	Heterogeneous nuclear ribonucleoproteins A2/B1 OS=Homo sapiens OX=9606 GN=HNRNPA2B1 PE=1 SV=2		1	
Q13835	Plakophilin-1 OS=Homo sapiens OX=9606 GN=PKP1 PE=1 SV=2		1	
O60506	Heterogeneous nuclear ribonucleoprotein Q OS=Homo sapiens OX=9606 GN=SYNCRIP PE=1 SV=2		1	
Q96DG6	Carboxymethylenebutenolidase homolog OS=Homo sapiens OX=9606 GN=CMBL PE=1 SV=1		1	
Q8N7H5	RNA polymerase II-associated factor 1 homolog OS=Homo sapiens OX=9606 GN=PAF1 PE=1 SV=2		1	
P09429	High mobility group protein B1 OS=Homo sapiens OX=9606 GN=HMGB1 PE=1 SV=3		1	
P04083	Annexin A1 OS=Homo sapiens OX=9606 GN=ANXA1 PE=1 SV=2		1	
Q2M2H8	Probable maltase-glucoamylase 2 OS=Homo sapiens OX=9606 GN=MGAM2 PE=2 SV=3		1	
P16402	Histone H1.3 OS=Homo sapiens OX=9606 GN=HIST1H1D PE=1 SV=2		1	
O14929	Histone acetyltransferase type B catalytic subunit OS=Homo sapiens OX=9606 GN=HAT1 PE=1 SV=1		1	
Q5D862	Filaggrin-2 OS=Homo sapiens OX=9606 GN=FLG2 PE=1 SV=1		1	
Q86V81	THO complex subunit 4 OS=Homo sapiens OX=9606 GN=ALYREF PE=1 SV=3		1	
P13489	Ribonuclease inhibitor OS=Homo sapiens OX=9606 GN=RNH1 PE=1 SV=2		1	
Q8IZ52	Chondroitin sulfate synthase 2 OS=Homo sapiens OX=9606 GN=CHPF PE=1 SV=2		1	
P57721	Poly(rC)-binding protein 3 OS=Homo sapiens OX=9606 GN=PCBP3 PE=2 SV=2		1	
Q6UWP8	Suprabasin OS=Homo sapiens OX=9606 GN=SBSN PE=1 SV=2		1	
Q5JUX0	Spindlin-3 OS=Homo sapiens OX=9606 GN=SPIN3 PE=1 SV=1		1	
P60900	Proteasome subunit alpha type-6 OS=Homo sapiens OX=9606 GN=PSMA6 PE=1 SV=1		1	

Supplementary Table 2. CD8+ T-cell infiltration-associated genes in TIMER Database

Gene	Spearman's rank correlation coefficient	Pvalue
ZNHIT2	-0.305403222	6.08E-25
ZNHIT1	-0.328126428	9.46E-29
ZNF692	-0.316025489	1.11E-26
ZNF579	-0.329228594	6.06E-29
ZNF444	-0.318258281	4.68E-27
ZNF205	-0.325767438	2.44E-28
ZMYND19	-0.321952541	1.10E-27
YIF1A	-0.30429471	9.15E-25
YDJC	-0.346710502	4.09E-32
WBSCR22	-0.309048103	1.57E-25
VPS72	-0.304036603	1.01E-24
VPS28	-0.345896971	5.80E-32
VGF	-0.326709072	1.67E-28
UQCC3	-0.398107349	1.13E-42
UQCC2	-0.422788248	1.88E-48
UFSP1	-0.314509265	1.98E-26
UBE2S	-0.340433325	5.94E-31
UBE2M	-0.333144395	1.23E-29
TUFM	-0.325542232	2.66E-28
TUBG1	-0.303623303	1.17E-24
TSR3	-0.344542593	1.04E-31
TSFM	-0.300355154	3.85E-24
TSEN54	-0.328597737	7.82E-29
TSEN34	-0.304786648	7.64E-25
TRMT61A	-0.338245607	1.49E-30
TRMT112	-0.353524196	2.09E-33
TRAPPC6A	-0.317830102	5.52E-27
TPRA1	-0.325886212	2.32E-28
TOP1MT	-0.333761355	9.55E-30
TOMM40	-0.337709266	1.86E-30
TMUB1	-0.339165438	1.01E-30
TMEM249	-0.313222946	3.24E-26
TMEM238	-0.315871427	1.18E-26
TMEM161A	-0.316401985	9.59E-27
TMEM147	-0.321988146	1.09E-27
TLCD1	-0.300222553	4.04E-24
TIMM17B	-0.402587645	1.09E-43
TIMM13	-0.355686215	8.00E-34
TIGD5	-0.375963442	6.85E-38
THOP1	-0.311832494	5.49E-26
TFAP4	-0.365860162	7.92E-36
TCEB2	-0.377918851	2.68E-38
TBRG4	-0.303247041	1.34E-24
TBL3	-0.318505617	4.25E-27
TBCB	-0.334855119	6.08E-30
SYNE4	-0.386964233	3.20E-40
SURF2	-0.338090395	1.59E-30
STRA13	-0.324424588	4.16E-28
SSNA1	-0.318972753	3.54E-27
SNRPB	-0.346740789	4.03E-32
SNRPA	-0.304859238	7.43E-25
SNRNP25	-0.312341882	4.53E-26
SMKR1	-0.326551491	1.78E-28
SLMO1	-0.328739647	7.39E-29
SLC39A4	-0.326847785	1.58E-28
SLC27A5	-0.333860145	9.17E-30
SLC25A39	-0.446569153	1.70E-54
SLC25A10	-0.368114441	2.78E-36
SLC19A1	-0.325775328	2.43E-28
SHARPIN	-0.365062048	1.14E-35

SAMD1	-0.31903595	3.46E-27
SAC3D1	-0.368305121	2.55E-36
RUVBL2	-0.395564415	4.17E-42
RUVBL1	-0.302081301	2.06E-24
RTKN	-0.305145924	6.69E-25
RRS1	-0.313760849	2.64E-26
RRP9	-0.385322127	7.22E-40
RRP1	-0.328604957	7.80E-29
RPUSD1	-0.376628168	4.98E-38
RPP21	-0.379946683	1.00E-38
RPL8	-0.316162724	1.05E-26
ROMO1	-0.34869932	1.73E-32
RNF208	-0.312772972	3.85E-26
RHPN1	-0.314960314	1.67E-26
RFNG	-0.319482291	2.91E-27
RECQL4	-0.334749864	6.35E-30
RCCD1	-0.313843465	2.56E-26
RBBP8NL	-0.366816136	5.09E-36
RAC3	-0.314451804	2.03E-26
PYCRL	-0.363298515	2.57E-35
PUS1	-0.321022293	1.59E-27
PTOV1	-0.351916214	4.24E-33
PTCD1	-0.357218835	4.03E-34
PSMG3	-0.327867609	1.05E-28
PSMD4	-0.32082254	1.72E-27
PRR7	-0.332764443	1.44E-29
PQBP1	-0.334212771	7.93E-30
PPP1R35	-0.383090513	2.17E-39
PPP1R16A	-0.372785092	3.11E-37
POP7	-0.434359504	2.47E-51
POLR2J	-0.387258419	2.76E-40
POLR2I	-0.391662841	3.03E-41
POLR2H	-0.369695597	1.33E-36
POLD2	-0.335492321	4.68E-30
PMVK	-0.337201952	2.30E-30
PKP3	-0.374554208	1.34E-37
PGP	-0.328649932	7.66E-29
PFDN6	-0.417759143	3.09E-47
PFDN2	-0.352163988	3.80E-33
PET100	-0.307607462	2.69E-25
PELP1	-0.3206534	1.84E-27
PDAP1	-0.349663953	1.14E-32
PAM16	-0.394809373	6.13E-42
PAFAH1B3	-0.350779876	6.98E-33
OXLD1	-0.391725973	2.94E-41
NUBP2	-0.338039368	1.62E-30
NTHL1	-0.380928261	6.23E-39
NT5M	-0.310909294	7.79E-26
NT5C3B	-0.340558393	5.63E-31
NT5C	-0.309120386	1.53E-25
NSUN5	-0.335406625	4.84E-30
NR2F6	-0.373220951	2.53E-37
NR2C2AP	-0.371141031	6.75E-37
NOP16	-0.326443631	1.86E-28
NOL3	-0.305479439	5.91E-25
NOC4L	-0.343762159	1.45E-31
NMRAL1	-0.354071636	1.64E-33
NME4	-0.313626043	2.78E-26
NME1	-0.362455048	3.78E-35
NHP2	-0.346376702	4.72E-32
NFKBIL1	-0.311000818	7.52E-26
NELFE	-0.351912688	4.25E-33
NDUFS8	-0.348761996	1.68E-32
NDUFB9	-0.314062009	2.35E-26
NDUFB7	-0.305478245	5.92E-25

NDUFB11	-0.319048675	3.44E-27
NDUFB10	-0.388785269	1.29E-40
NDUFAF3	-0.31340127	3.03E-26
NDUFA7	-0.380615699	7.26E-39
NDUFA13	-0.310168278	1.03E-25
NDUFA11	-0.312781365	3.83E-26
NAT9	-0.318131416	4.92E-27
NARFL	-0.314054417	2.36E-26
NAA38	-0.306725131	3.73E-25
NAA10	-0.334916734	5.93E-30
MZT2B	-0.311239586	6.87E-26
MRPS34	-0.395418829	4.49E-42
MRPS26	-0.40418488	4.71E-44
MRPS12	-0.310394322	9.46E-26
MRPL55	-0.362036513	4.58E-35
MRPL52	-0.308955177	1.62E-25
MRPL28	-0.339462592	8.93E-31
MRPL24	-0.396850999	2.15E-42
MRPL23	-0.360628392	8.68E-35
MRPL17	-0.316137653	1.06E-26
MRPL14	-0.405951539	1.85E-44
MRPL12	-0.385883021	5.47E-40
MLST8	-0.345154943	7.98E-32
MLF2	-0.362279361	4.10E-35
MIR210HG	-0.302557338	1.73E-24
MFSD3	-0.393114099	1.45E-41
MEA1	-0.300242395	4.01E-24
MBLAC1	-0.339404623	9.15E-31
MAGIX	-0.366364721	6.27E-36
MAFG-AS1	-0.331129003	2.80E-29
MACROD1	-0.339277908	9.65E-31
LSM4	-0.402172578	1.36E-43
LRRC45	-0.332306686	1.73E-29
KRTCAP3	-0.377820846	2.81E-38
KRTCAP2	-0.353749142	1.89E-33
KLF16	-0.313345274	3.09E-26
KIFC2	-0.351772499	4.52E-33
KCTD13	-0.309327215	1.41E-25
KAT2A	-0.371845232	4.85E-37
IPO4	-0.310698013	8.43E-26
ICT1	-0.330250772	4.01E-29
HSPBP1	-0.364563104	1.44E-35
HSPA1B	-0.326035315	2.19E-28
HSPA1A	-0.302410691	1.83E-24
HSF4	-0.304073205	9.93E-25
HSF1	-0.312417808	4.40E-26
HSD17B10	-0.305493379	5.88E-25
HRAS	-0.331910228	2.04E-29
HGH1	-0.331437797	2.47E-29
HAGHL	-0.31025303	9.97E-26
HAGH	-0.330019249	4.40E-29
GTPBP3	-0.330839055	3.16E-29
GPS1	-0.306674728	3.80E-25
GLI4	-0.333468732	1.08E-29
GADD45GIP1	-0.357862178	3.02E-34
FN3K	-0.34428892	1.16E-31
FDX1L	-0.366249324	6.62E-36
FBXL6	-0.31781608	5.55E-27
FBF1	-0.312019262	5.12E-26
FARSA	-0.333675496	9.89E-30
FAM50A	-0.336859317	2.65E-30
FAM3A	-0.300091543	4.24E-24
FAM195A	-0.422457592	2.26E-48
FAM173A	-0.31366335	2.74E-26
EXOSC5	-0.375292822	9.44E-38

EXOSC4	-0.397137783	1.86E-42
ENKD1	-0.317355714	6.64E-27
EMC9	-0.318422247	4.39E-27
ELMO3	-0.324135975	4.66E-28
EIF6	-0.32555906	2.65E-28
ECI1	-0.340901368	4.87E-31
DYNLRB1	-0.333377872	1.12E-29
DUS1L	-0.346875655	3.81E-32
DRAP1	-0.307002657	3.36E-25
DPM3	-0.362014899	4.62E-35
DOHH	-0.314841847	1.75E-26
DNPH1	-0.338361737	1.42E-30
DHPS	-0.302217447	1.96E-24
DECR2	-0.304099629	9.83E-25
DDX56	-0.300582174	3.55E-24
CYHR1	-0.3615961	5.59E-35
CYC1	-0.335861824	4.01E-30
CUTA	-0.406449363	1.42E-44
CTU1	-0.338903099	1.13E-30
COX6A1	-0.313323469	3.12E-26
COX17	-0.347513551	2.89E-32
COPS6	-0.335600107	4.47E-30
COMTD1	-0.343556447	1.58E-31
COMMD5	-0.308043887	2.28E-25
COMMD4	-0.33182195	2.11E-29
COA6	-0.308558631	1.88E-25
CLPP	-0.32239657	9.27E-28
CHTF18	-0.328542156	8.00E-29
CHCHD1	-0.315988322	1.12E-26
CEP131	-0.300930047	3.13E-24
CDK5	-0.32437825	4.23E-28
CCDC167	-0.313632845	2.77E-26
CCDC124	-0.323522346	5.95E-28
C8orf82	-0.352076338	3.95E-33
C7orf55	-0.311377802	6.52E-26
C1orf35	-0.387461917	2.50E-40
C19orf73	-0.316391475	9.63E-27
C19orf53	-0.303857227	1.07E-24
C17orf89	-0.338824361	1.17E-30
C16orf59	-0.358677681	2.09E-34
C16orf13	-0.448484659	5.29E-55
C12orf73	-0.332384804	1.68E-29
C10orf35	-0.304728452	7.80E-25
BRMS1	-0.301100844	2.94E-24
BOP1	-0.355922685	7.20E-34
BOLA1	-0.399879554	4.49E-43
BANF1	-0.344455306	1.08E-31
B4GALNT4	-0.306433146	4.15E-25
B3GAT3	-0.315845156	1.19E-26
AURKAIP1	-0.312704639	3.95E-26
ATP6V0B	-0.310237914	1.00E-25
ATP5J2	-0.307159995	3.17E-25
ATP5I	-0.314874302	1.72E-26
ATP5D	-0.305325839	6.26E-25
ARTN	-0.319277174	3.15E-27
APOA1BP	-0.359544076	1.42E-34
AP1M2	-0.301774721	2.30E-24
ANKRD39	-0.319884039	2.49E-27
ANAPC11	-0.389332649	9.80E-41
ALKBH2	-0.314010512	2.40E-26
ADCK5	-0.319139311	3.32E-27

Supplementary Table 3. The correlation between clinicopathological parameters and TMUB1/HUWE1/STT3A expression in breast cancer.

	TMUB1 Low, n(%)	High, n(%)	*P**
**Age**			
< 45	15(48.3)	16(51.7)	0.828
≥45	35(50.7)	34(49.3)	
**ER status**			
Negative	18(56.3)	14(43.7)	0.391
Positive	32(47.1)	36(52.9)	
**PR status**			
Negative	23(56.1)	18(43.9)	0.309
Positive	27(45.7)	32(54.3)	
**HER2 status**			
Negative	23(48.9)	24(51.1)	0.841
Positive	27(50.9)	26(49.1)	
**Lymph node invasion**			
Absent	25(61.0)	16(39.0)	0.067
Present	25(42.4)	34(57.6)	
**Pathological subtype**			
Ductal	48(51.1)	46(48.9)	0.241
Lobular	1(100)	0(0.00)	
Others	1(20.0)	4(80.0)	
**TNM stage**			
I-II	32(51.6)	30(48.4)	0.68
III-IV	18(47.4)	20(52.6)	

**P* values determined by Chi-square

	HUWE1 Low, n(%)	High, n(%)	*P**
**Age**			
< 45	12(38.7)	19(61.3)	0.130
≥45	38(55.1)	31(44.9)	
**ER status**			
Negative	16(50)	16(50)	1.00
Positive	34(50)	34(50)	
**PR status**			
Negative	20(48.8)	21(51.2)	0.839
Positive	30(50.8)	29(49.2)	
**HER2 status**			
Negative	20(42.6)	27(57.4)	0.161
Positive	30(56.6)	23(43.4)	
**Lymph node invasion**			
Absent	23(56.1)	18(43.9)	0.309
Present	27(45.8)	32(54.2)	
**Pathological subtype**			
Ductal	48(51.1)	46(48.9)	0.537
Lobular	0(0)	1(100)	
Others	2(40.0)	3(60.0)	
**TNM stage**			
I-II	28(54.2)	34(54.8)	0.216
III-IV	22(57.9)	16(42.1)	

**P* values determined by Chi-square

	STT3A Low, n(%)	High, n(%)	*P**
**Age**			
< 45	17(54.8)	14(45.2)	0.517
≥45	33(47.8)	36(52.2)	
**ER status**			
Negative	13(40.6)	19(59.4)	0.198
Positive	37(54.4)	31(45.6)	
**PR status**			
Negative	25(61.0)	16(39.0)	0.067
Positive	25(42.4)	34(57.6)	
**HER2 status**			
Negative	21(44.7)	26(55.3)	0.316
Positive	29(54.7)	24(45.3)	
**Lymph node invasion**			
Absent	21(51.2)	20(48.8)	0.839
Present	29(49.2)	30(50.8)	
**Pathological subtype**			
Ductal	47(50.0)	47(50.0)	0.549
Lobular	1(100)	0(0.00)	
Others	2(40.0)	3(60.0)	
**TNM stage**			
I-II	30(48.4)	32(51.6)	0.68
III-IV	20(52.6)	18(47.4)	

**P* values determined by Chi-square

Supplementary Table 4. The correlation between clinicopathological parameters and TMUB1/HUWE1/STT3A expression in gastric cancer.

	TMUB1		
	**Low, n(%)**	**High, n(%)**	***P****
**Age**			
< 45	7(43.8)	9(56.2)	0.571
≥45	29(51.8)	27(48.2)	
**Gender**			
Male	19(45.2)	23(54.8)	0.339
Female	17(56.7)	13(43.3)	
**Invasive depth**			
T1-T2	13(61.9)	8(38.1)	0.195
T3-T4	23(45.1)	28(54.9)	
**Lymph node metastasis**			
Absent	19(46.3)	22(53.7)	0.475
Present	17(54.8)	14(45.2)	
**TNM Stage**			
I-II	17(54.8)	14(45.2)	0.475
III-IV	19(46.3)	22(53.7)	
**P* values determined by Chi-square			

	HUWE1		
	**Low, n(%)**	**High, n(%)**	***P****
**Age**			
< 45	8(50.0)	8(50.0)	1.00
≥45	28(50.0)	28(50.0)	
**Gender**			
Male	24(57.1)	18(42.9)	0.151
Female	12(40.0)	18(60.0)	
**Invasive depth**			
T1-T2	7(33.3)	14(66.7)	0.13
T3-T4	25(53.2)	22(46.8)	
**Lymph node metastasis**			
Absent	17(41.5)	24(58.5)	0.096
Present	19(61.3)	12(38.7)	
**TNM Stage**			
I-II	12(38.7)	19(61.3)	0.096
III-IV	24(58.5)	17(41.5)	
**P* values determined by Chi-square			

	STT3A		
	**Low, n(%)**	**High, n(%)**	***P****
**Age**			
< 45	10(62.5)	6(37.5)	0.257
≥45	26(46.4)	30(53.6)	
**Gender**			
Male	18(42.9)	24(57.1)	0.151
Female	18(60.0)	12(40.0)	
**Invasive depth**			
T1-T2	10(47.6)	11(52.4)	0.795
T3-T4	26(51.0)	25(49.0)	
**Lymph node metastasis**			
Absent	18(43.9)	23(56.1)	0.234
Present	18(58.1)	13(41.9)	
**TNM Stage**			
I-II	15(48.4)	16(51.6)	0.812
III-IV	21(51.2)	20(48.8)	
**P* values determined by Chi-square			

Supplementary Table 5. Sequences of Oligonucleotides for qPCR and RNA Interference.

Oligonucleotides Resource	Sequence	Application
Sh-hTMUB1#1-F	5'-CCGGGGCTGAAATTCCTCAATGATTCTCGAGAATCATTGAGGAATTTCAGCCTTTTTG-3'	RNA interference
Sh-hTMUB1#1-R	5'-AATTCAAAAAGGCTGAAATTCCTCAATGATTCTCGAGAATCATTGAGGAATTTCAGCC-3'	RNA interference
Sh-hTMUB1#2-F	5'-CCGGCTACGGCTGAAATTCCTCATTCTCGAGAATGAGGAATTTCAGCCGTAGTTTTTG-3'	RNA interference
Sh-hTMUB1#2-R	5'-AATTCAAAAACTACGGCTGAAATTCCTCATTCTCGAGAATGAGGAATTTCAGCCGTAG-3'	RNA interference
sgRNA-hTMUB1 F #4	5'-caccgAACGCACACCGCTGAGGGCG-3'	gRNA for TMUB1-KO
sgRNA-hTMUB1 R #4	5'-aaacCGCCCTCAGCGGTGTGCGTTc-3'	gRNA for TMUB1-KO
sgRNA-hCD274 F	5'-caccgGGTTCCCAAGGACCTATATG-3'	gRNA for CD274-KO
sgRNA-hCD274 R	5'-aaacCATATAGGTCCTTGGGAACCc-3'	gRNA for CD274-KO
sgRNA-mCd274 F	5'-caccgGCCTGCTGTCACTTGCTACG-3'	gRNA for Cd274-KO
sgRNA-mCd274 R	5'-aaacCGTAGCAAGTGACAGCAGGCc-3'	gRNA for Cd274-KO
sh-mTMUB1#1-F	5'-CCGGCAATGACTCTGAGCAGGTATTCTCGAGAATACCTGCTCAGAGTCATTGTTTTTG-3'	RNA interference
sh-mTMUB1#1-R	5'-AATTCAAAAACAATGACTCTGAGCAGGTATTCTCGAGAATACCTGCTCAGAGTCATTG-3'	RNA interference
sh-mTMUB1#2-F	5'-CCGGGGTTGAAATTTCTCAATGATTCTCGAGAATCATTGAGAAATTTCAACCTTTTTG-3'	RNA interference
sh-mTMUB1#2-R	5'-AATTCAAAAAGGTTGAAATTTCTCAATGATTCTCGAGAATCATTGAGAAATTTCAACC-3'	RNA interference
sh-hHUWE1-F	5'-CCGGCCACACTTTCACAGATACTATCTCGAGATAGTATCTGTGAAAGTGTGGTTTTTG-3'	RNA interference
sh-hHUWE1-R	5'-AATTCAAAAACCACACTTTCACAGATACTATCTCGAGATAGTATCTGTGAAAGTGTGG-3'	RNA interference
sh-Scramble-F	5'-CCGGGTACTGCAGCCACATTCTCCCTCGAGGAGAATGTGGCTGCAGTCTTTTTG-3'	RNA interference
sh-Scramble-R	5'-AATTCAAAAAGTACTGCAGCCACATTCTCCTCGAGGAGAATGTGGCTGCAGTAC-3'	RNA interference
hPD-L1-F	5'-ACAGCTGAATTGGTCATCCC-3'	RT-qPCR primer
hPD-L1-R	5'-TGTCAGTGCTACACCAAGGC-3'	RT-qPCR primer
hTMUB1-F	5'-AGTTTAGGAGAGGCGTTGCT-3'	RT-qPCR primer
hTMUB1-R	5'-TGAGCTCGAGGTCTTCAACC-3'	RT-qPCR primer
hIFNγ-F	5'-TCGGTAACTGACTTGAATGTCCA-3'	RT-qPCR primer
hIFNγ-R	5'-TCGCTTCCCTGTTTTAGCTGC-3'	RT-qPCR primer
hTNFα-F	5'-CCTCTCTCTAATCAGCCCTCTG-3'	RT-qPCR primer
hTNFα-R	5'-GAGGACCTGGGAGTAGATGAG-3'	RT-qPCR primer
hGAPDH-F	5'-AAGGTGAAGGTCGGAGTCAA-3'	RT-qPCR primer
hGAPDH-R	5'-AATGAAGGGGTCATTGATGG-3'	RT-qPCR primer
mActinβ-F	5’-GGCTGTATTCCCCTCCATCG-3’	RT-qPCR primer
mActinβ-R	5’-CCAGTTGGTAACAATGCCATGT-3’	RT-qPCR primer
mIFNγ-F	5’-ACAGCAAGGCGAAAAAGGATG-3’	RT-qPCR primer
mIFNγ-R	5’-TGGTGGACCACTCGGATGA-3’	RT-qPCR primer
mTNFα-F	5’-CCCTCACACTCAGATCATCTTCT-3’	RT-qPCR primer
mTNFα-R	5’-GCTACGACGTGGGCTACAG-3’	RT-qPCR primer
mCCL5-F	5’-GCTGCTTTGCCTACCTCTCC-3’	RT-qPCR primer
mCCL5-R	5’-TCGAGTGACAAACACGACTGC-3’	RT-qPCR primer
mCXCL10-F	5’-TGAATCCGGAATCTAAGACCATCAA-3’	RT-qPCR primer
mCXCL10-R	5’-AGGACTAGCCATCCACTGGGTAAAG-3’	RT-qPCR primer
hSTT3A-F	5'-AACAGCTGCAAACCCTGAGA-3'	RT-qPCR primer
hSTT3A-R	5'-GGCCACATAGATCCGGTGAG-3'	RT-qPCR primer
hHUWE1-F	5'-ACTGGTGCAACTTCCTCCTT-3'	RT-qPCR primer
hHUWE1-R	5'-TGGTCTTCTGGCGACCTAGT-3'	RT-qPCR primer
hCMTM4-F	5'-CAGTGCGTTTGTGGTGACTG-3'	RT-qPCR primer
hCMTM4-R	5'-GGAAAAAGTGTCCAGGCGCT-3'	RT-qPCR primer
hCMTM6-F	5'-AAGCTCCGCTTGTTCTGAGG-3'	RT-qPCR primer
hCMTM6-R	5'-TTCTGGTCCCAAGTTCCCCT-3'	RT-qPCR primer

Supplementary Table 6. Antibody Information

ANTIBODY	SOURCE	IDENTIFIER	DILUTIONS
anti-TMUB1	abcam	Cat#ab180586	1:2000 for IB, 1:400 for IF and 1:400 for IHC
anti-HUWE1	abcam	Cat#ab70161	1:2000 for IB and 1:400 for IF
anti-Calnexin	abcam	Cat#ab112995	1:400 for IF
anti-PD-L1(Alexa Fluor 488)	abcam	Cat#ab209959	1:500 for FC
anti-PD-L1(Alexa Fluor 647)	abcam	Cat#ab209960	1:500 for FC
Isotype Control (Alexa Fluor 488 Rabbit IgG)	abcam	Cat#ab199091	1:500 for FC
Isotype Control (Alexa Fluor 647 Rabbit IgG)	abcam	Cat#ab199093	1:500 for FC
goat anti-rabbit IgG H&L(Alexa Fluor 488)	abcam	Cat#ab150077	1:400 for IF
goat anti-rabbit IgG H&L(Alexa Fluor 594)	abcam	Cat#ab150080	1:400 for IF
goat anti-mouse IgG H&L(Alexa Fluor 488)	abcam	Cat#ab150113	1:400 for IF
goat anti-mouse IgG H&L(Alexa Fluor 594)	abcam	Cat#ab150116	1:400 for IF
anti-LAMP1	Cell Signaling Technology	Cat#9091	1:50 for IF
anti-Lamin B1	Cell Signaling Technology	Cat#12586	1:2000 for IB
anti-Vinculin	Cell Signaling Technology	Cat#13901	1:2000 for IB
anti-Ubiquitin	Cell Signaling Technology	Cat#58395	1:2000 for IB
anti-Tom20	Cell Signaling Technology	Cat#42406	1:2000 for IB
anti-Calnexin	Cell Signaling Technology	Cat#2679	1:2000 for IB
anti-αTubulin	Cell Signaling Technology	Cat#2125	1:2000 for IB
anti-His-tag	Abmart	Cat#M20001	1:5000 for IB
anti-Myc-tag	Abmart	Cat#M20002	1:5000 for IB
anti-DYKDDDDK-tag	Abmart	Cat#M20008	1:5000 for IB
anti-HA-tag	Abmart	Cat#M20003	1:5000 for IB
anti-GAPDH	Abmart	Cat#M20050	1:5000 for IB
HRP goat anti-mouse IgG	Bioker	Cat#BK-M050	1:5000 for IB
HRP goat anti-rabbit IgG	Bioker	Cat#BK-R050	1:5000 for IB
anti-PD-L1	Proteintech	Cat#17952-1-AP	1:1000 for IB,1:50 for IP
anti-PD-L1	Proteintech	Cat#66248-1-Ig	1:400 for IF,1:200 for IHC
anti-CD8-APC	Proteintech	Cat#APC-65069	1:500 for FC
anti-CD3-APC-A750	eBioscience	Cat#47-0032	1:500 for FC
anti-GzmB-FITC	eBioscience	Cat#11-8898	1:500 for FC
anti-CD3	eBioscience	Cat#16-0037	100 ng/ml for T cell killing assay
anti-CD4-PE	eBioscience	Cat#12-0041	1:500 for FC
anti-Foxp3-APC	eBioscience	Cat#17-5773	1:500 for FC
anti-CD11b-PE	eBioscience	Cat#12-0112	1:500 for FC
anti-CD45-PB450	Biolegend	Cat#103126	1:500 for FC
anti-CD3-APC	Biolegend	Cat#100235	1:500 for FC
anti-CD8α-APC	Biolegend	Cat#300912	1:500 for FC
anti-TIM3-PE	Biolegend	Cat#345006	1:500 for FC
anti-NK1.1-PE	Biolegend	Cat#108707	1:500 for FC
anti-F/480-APC	Biolegend	Cat#123116	1:500 for FC
anti-Gr1-APC	Biolegend	Cat#108424	1:500 for FC
InVivoMAb anti-mouse CTLA-4	Bio X Cell	Cat#BE0032	10 mg/kg for *in vivo* use
InVivoMAb anti-mouse NK1.1	Bio X Cell	Cat#BE0036	10 mg/kg for *in vivo* use
InVivoMAb anti-mouse CD8α	Bio X Cell	Cat#BE0117	10 mg/kg for *in vivo* use
InVivoMAb anti-mouse CD4	Bio X Cell	Cat#BE0003-1	10 mg/kg for *in vivo* use
anti-ITM1	SANTA CRUZ	Cat#sc-390227	1:1000 for IB
anti-CMTM4	HUABIO	Cat#ER63053	1:1000 for IB
anti-CMTM6	HUABIO	Cat#ER65542	1:1000 for IB

Supplementary Table 7. Reagent and Resource

REAGENT or RESOURCE	SOURCE	IDENTIFIER
**Bacterial and Virus Strains**		
DH5a Competent Cells	Thermo Fisher Scientifific	Cat#18265017
BL21-CodonPlus (DE3)-RP-X Competent cells	Agilent	Cat#260275
**Biological Samples**		
Fresh frozen breast cancer tissues	Sun Yat-sen University Cancer Center	See Supplementary Table 3
Fresh frozen gastric cancer tissues	The Second Affiliated Hospital of Zhejiang University	See Supplementary Table 4
**Chemicals, Peptides, and Recombinant Proteins**		
MBP-TMUB1-His	This paper	N/A
GST-PD-L1	This paper	N/A
PTPR	This paper	N/A
PTPR-Biotin	This paper	N/A
PTPR-FITC	This paper	N/A
IL-2	PeproTech	Cat#200-02
MG132	MedChemExpress	Cat#HY-13259
Tunicamycin	MedChemExpress	Cat#HY-A0098
Chloroquine	MedChemExpress	Cat#HY-17589A
Eeyarestatin I	APExBio	Cat#B7535
3X Flag peptide	APExBio	Cat#A6001
MTT	APExBio	Cat#B7777
IFN-γ	PeproTech	Cat#AF-300-02
PNGase-F	Mei5 Biotechnology	Cat#MF462
Percoll	SOLARBIO	Cat#P8370
**Critical Commercial Assays**		
Zombie Violet™ Fixable Viability Kit	BioLegend	Cat#423113
Histostain-Plus IHC Kit, Rabbit Primary	NeoBioscience	Cat#ENS004
QuantiCyto® Mouse IFN-γ ELISA kit	NeoBioscience	Cat#EMC101g
Human CD3/CD28/CD2 T cell activator	STEMCELL Technologies	Cat#10970
TUBE 2 Agarose beads	LifeSensors	Cat#UM402
ClonExpress II One Step Cloning Kit	Vazyme	Cat#C112
eBioscience^{TM} Fixable Viability Dye eFluor^{TM} 520	eBioscience	Cat#65-0867
Foxp3 / Transcription Factor Staining Buffer Set	eBioscience	Cat#00-5523
**Experimental Models: Organisms/Strains**		
Five-week BALB/c female nude mice	Shanghai Laboratory Animals Center	N/A
Five-week BALB/c female mice	Shanghai Laboratory Animals Center	N/A
Five-week C57BL/6 female mice	Shanghai Laboratory Animals Center	N/A
**Oligonucleotides**		
Oligonucleotides for qPCR, RNA interference	This paper	See Supplementary Table 5
**Software and Algorithms**		
GraphPad Prism Software	GraphPad software	https://www.graphpad.com/
Leica Application Suite X	Leica Microsystems	https://www.leica-microsystems.com
ImageJ Fiji	National Institutes of Health	https://fiji.sc/
