# Supplementary material for: Promoting anti-tumor immunity by targeting TMUB1 to modulate PD-L1 polyubiquitination and glycosylation
Source: Nat Commun. 2022 Nov 14;13:6951. doi: 10.1038/s41467-022-34346-x (PMC9663433; doi:10.1038/s41467-022-34346-x)
Supplement: Supplementary file 3 — Reporting Summary [file 41467_2022_34346_MOESM3_ESM.pdf]

nature portfolio

Corresponding author(s): Aifu Lin, Tianhua Zhou, Ruhong Zhou, Wenqi Wang, Xu Li,

Last updated by author(s): Oct 7, 2022

Reporting Summary

Nature Portfolio wishes to improve the reproducibility of the work that we publish. This form provides structure for consistency and transparency in reporting. For further information on Nature Portfolio policies, see our Editorial Policies and the Editorial Policy Checklist.

Statistics

For all statistical analyses, confirm that the following items are present in the figure legend, table legend, main text, or Methods section.

n/a	Confirmed	
☐	☒	The exact sample size (*n*) for each experimental group/condition, given as a discrete number and unit of measurement
☐	☒	A statement on whether measurements were taken from distinct samples or whether the same sample was measured repeatedly
☐	☒	The statistical test(s) used AND whether they are one- or two-sided<br>*Only common tests should be described solely by name; describe more complex techniques in the Methods section.*
☒	☐	A description of all covariates tested
☐	☒	A description of any assumptions or corrections, such as tests of normality and adjustment for multiple comparisons
☐	☒	A full description of the statistical parameters including central tendency (e.g. means) or other basic estimates (e.g. regression coefficient) AND variation (e.g. standard deviation) or associated estimates of uncertainty (e.g. confidence intervals)
☐	☒	For null hypothesis testing, the test statistic (e.g. *F*, *t*, *r*) with confidence intervals, effect sizes, degrees of freedom and *P* value noted<br>*Give P values as exact values whenever suitable.*
☒	☐	For Bayesian analysis, information on the choice of priors and Markov chain Monte Carlo settings
☒	☐	For hierarchical and complex designs, identification of the appropriate level for tests and full reporting of outcomes
☐	☒	Estimates of effect sizes (e.g. Cohen's *d*, Pearson's *r*), indicating how they were calculated

Our web collection on statistics for biologists contains articles on many of the points above.

Software and code

Policy information about availability of computer code

Data collection	Commercial softwares equipped by CFX96 real-time PCR (CFX Manager, Bio-Rad), ChemiDoc Touch Imaging System (Bio-Rad), Infinite M200 Pro(Tecan), FV3000 confocal microscope (Olympus), Super resolution Confocal Laser scanning microscope TCS SP8 STED (Leica), UltiMate 3000 RSLC nano System (Thermo Fisher Scientific), Orbitrap Fusion Lumos mass spectrometer (Thermo Fisher Scientific).
Data analysis	Image Lab version 4.1 (Bio-Rad) was used to acquire Immunoblots and protein Coomassie staining gels. Acquired raw images were analyzed using FV31S-SW Viewer version 2.3.1 (Olympus), FV31S-DT version 2.3.1 (Olympus) and Leica Application Suite X version 3.3.0.16799 (Leica). The quantification of IHC staining density was measured using Fiji Software version 2.3.0 (ImageJ, NIH) software. Statistical analysis was performed with GraphPad Prism version 8.0.2 (GraphPad Software, Inc.), Mascot software program version 2.7.0 (Matrix Science, Boston, MA). differentially expressed RNAs between tumor and non-malignant samples from breast cancer were analyzed by DESeq2 (R package (1.16.1)). ClusterProflier (R package (4.1.4)) was utilized to perform GO analysis.

For manuscripts utilizing custom algorithms or software that are central to the research but not yet described in published literature, software must be made available to editors and reviewers. We strongly encourage code deposition in a community repository (e.g. GitHub). See the Nature Portfolio guidelines for submitting code & software for further information.

Data

Policy information about availability of data

All manuscripts must include a data availability statement. This statement should provide the following information, where applicable:

- Accession codes, unique identifiers, or web links for publicly available datasets
- A description of any restrictions on data availability
- For clinical datasets or third party data, please ensure that the statement adheres to our policy

The PD-L1-IP mass spectrometry proteomics data have been deposited to the ProteomeXchange Consortium via the PRIDE partner repository with the dataset identifier PXD031702. All data are included in the Supplemental Information or available from the authors upon reasonable requests

Human research participants

Policy information about studies involving human research participants and Sex and Gender in Research.

Reporting on sex and gender	In this study, patients in this study were differentiated by biological sex, in which all breast cancer patients were female, while the gender composition of gastric cancer patients was reported in Supplementary Table 4. Only female patients were considered in all parts of this study involving breast cancer. The part of this study involving gastric cancer did not design the experiment for gender or sex.
Population characteristics	Information about breast and gastric cancer patients is included in Supplementary Table 3 and Supplementary Table 4, including age, gender of both cancers and HER2\ER\PR positivity of breast cancer patients.
Recruitment	All samples were collected from patients with informed consent, and all related procedures were performed with the approval of the internal review and ethics boards of SYSUCC or Ethics Committee of the Second Affiliated Hospital, School of Medicine Zhejiang University. Patients were recruited with no perceived bias. All patients were not treated with adjuvant radiotherapy or chemotherapy before operation.All patients were provided with informed written consents for obtaining study specimens.
Ethics oversight	The internal review and ethics boards of SYSUCC, Ethics Committee of the Second Affiliated Hospital, School of Medicine Zhejiang University

Note that full information on the approval of the study protocol must also be provided in the manuscript.

Field-specific reporting

Please select the one below that is the best fit for your research. If you are not sure, read the appropriate sections before making your selection.

☒ Life sciences ☐ Behavioural & social sciences ☐ Ecological, evolutionary & environmental sciences

For a reference copy of the document with all sections, see nature.com/documents/nr-reporting-summary-flat.pdf

Life sciences study design

All studies must disclose on these points even when the disclosure is negative.

Sample size	No statistical method was used to predetermine sample size. Sample size was chosen based on previous experience and standards in the field. All of the experiments were repeated least 3 times. The sample size for in vitro (n>=3) and in vivo (n>=5) are typical in the field. We determined the sample size based on previous published studies in the field over the past years (Aifu Lin, et al., Nat Cell Biol. 2016; Aifu Lin, et al., Nat Cell Biol, 2017; Xin Zheng, et al. EMBO J, 2017; Lingjie Sang, et al., Mol Cell, 2018; Lingjie Sang, et al., Nat Metab, 2021).
Data exclusions	There are no data exclusions.
Replication	For each representative image/data, experiments were performed at least three times with similar results unless otherwise noted in the manuscript.
Randomization	Samples and organisms were randomly allocated to experimental groups. No specific randomization protocol has been used. Mice were ageand sex matched.
Blinding	No specific blinding was applied since all experiments were assigned into groups including relevalant controls and analysis was done objectively and without bias.

Reporting for specific materials, systems and methods

We require information from authors about some types of materials, experimental systems and methods used in many studies. Here, indicate whether each material, system or method listed is relevant to your study. If you are not sure if a list item applies to your research, read the appropriate section before selecting a response.

Materials & experimental systems

n/a	Involved in the study
☐	☒ Antibodies
☐	☒ Eukaryotic cell lines
☒	☐ Palaeontology and archaeology
☐	☒ Animals and other organisms
☒	☐ Clinical data
☒	☐ Dual use research of concern

Methods

n/a	Involved in the study
☒	☐ ChIP-seq
☐	☒ Flow cytometry
☒	☐ MRI-based neuroimaging

Antibodies

Antibodies used

anti-TMUB1 (abcam, ab180586, Rb)[EPR14066]
anti-HUWE1 (abcam, Rb, ab70161)
anti-Calnexin (abcam, Ms, ab112995)[6F12BE10]
anti-PD-L1 (Alexa Fluor 488) (abcam, ab209959, Rb) [28-8]
anti-PD-L1 (Alexa Fluor 647) (abcam, ab209960, Rb) [28-8]
Isotype Control (Alexa Fluor 488 Rabbit IgG) (abcam, ab199091, Rb) [EPR25A]
Isotype Control (Alexa Fluor 647 Rabbit IgG) (abcam, ab199093, Rb) [EPR25A]
goat anti-rabbit IgG H&L(Alexa Fluor 488) (abcam, ab150077)
goat anti-rabbit IgG H&L(Alexa Fluor 594) (abcam, ab150080)
goat anti-mouse IgG H&L(Alexa Fluor 488) (abcam, ab150113)
goat anti-mouse IgG H&L(Alexa Fluor 594) (abcam, ab150116)
anti-LAMP1 (Cell Signaling Technology, 9091, Rb) [D2D11]
anti-Lamin B1 (Cell Signaling Technology, 12586, Rb) [D4Q4Z]
anti-Vinculin (Cell Signaling Technology, 13901, Rb) [E1E9V]
anti-ubiquitin (Cell Signaling Technology, 58395, Rb)
anti-TOM20 (Cell Signaling Technology, 42406, Rb) [D8T4N]
anti-Calnexin (Cell Signaling Technology, 2679, Rb) [C5C9]
anti-αTubulin (Cell Signaling Technology, 2125, Rb) [11H10]
anti-His-Tag (Abmart, M20001, Ms) [2A8]
anti-Myc-Tag (Abmart, M20002, Ms) [19C2]
anti-HA-Tag (Abmart, M20003, Ms) [26D11]
anti-DYKDDDDK-Tag (Abmart, M20008, Ms) [3B9]
anti-GAPDH (Abmart, M20050, Ms) [3F10]
HRP goat anti-mouse IgG (Bioker, BK-M050)
HRP goat anti-rabbit IgG (Bioker, BK-R050)
anti-PD-L1 (Proteintech, 17952-1-AP, Rb)
anti-PD-L1 (Proteintech, 66248-1-Ig, Ms) [2B11D11]
anti-CD8-APC (Proteintech, APC-65069, Rat) [53-6.7]
anti-CD3-APC-A750 (eBioscience, 47-0032, Rat) [17A2]
anti-GzmB-FITC (eBioscience, 11-8898, Rat) [NGZB]
anti-CD3 (eBioscience, 16-0037, Ms) [OKT3]
anti-CD4-PE (eBioscience, 12-0041, Rat) [GK1.5]
anti-Fxop3-APC (eBioscience, 17-5773, Rat) [FJK-16s]
anti-CD11b-PE (eBioscience, 12-0112, Rat) [M1/70]
anti-CD45-PB450 (Biolegend, 103126, Rat) [30-F11]
anti-CD3-APC (Biolegend, 100235, Rat) [17A2]
anti-CD8α-APC (Biolegend, 300912, Rat) [HIT8a]
anti-TIM3-PE (Biolegend, 345006, Rat) [F38-2E2]
anti-NK1.1-PE (Biolegend, 108707, Rat) [PK136]
anti-F/480-APC (Biolegend, 123116, Rat) [BM8]
anti-Gr1-APC (Biolegend, 108424, Rat) [RB6-8C5]
InVivoMAb anti-mouse CTLA-4 (Bio X Cell, BE0032) [UC10-4F10-11]
InVivoMAb anti-mouse NK1.1 (Bio X Cell, BE0036) [PK136]
InVivoMAb anti-mouse CD8α (Bio X Cell, BE0117) [YTS 169.4]
InVivoMAb anti-mouse CD4 (Bio X Cell, BE0003-1) [GK1.5]
anti-ITM1 (SANTA CRUZ, sc-390227, Ms) [A-2]
anti-CMTM4 (Huabio, ER63053, Rb)
anti-CMTM6 (Huabio, ER65542, Rb)

Validation

All antibodies used in this study are commercially available and all are validated by the vendors for the specific assays and species used; the validation data is available on the vendors website. As stated below, some antibodies are additionally validated in our

study.
1) anti-TMUB1 (abcam, ab180586, Rb)[EPR14066]:
https://www.abcam.cn/tmub1-antibody-epr14066-ab180586.html.
The manufacturer has validated this antibody for WB, IF and IHC in the species human.
2) anti-HUWE1 (abcam, Rb, ab70161):
https://www.abcam.cn/huwe1mule-antibody-ab70161.html
The manufacturer has validated this antibody for WB, IP and IF in the species human.
3) anti-Calnexin (abcam, Ms, ab112995)[6F12BE10]:
https://www.abcam.cn/calnexin-antibody-6f12be10-ab112995.html
The manufacturer has validated this antibody for IF in the species human.
4) anti-PD-L1 (Alexa Fluor 488) (abcam, ab209959, Rb) [28-8]:
https://www.abcam.cn/alexa-fluor-488-pd-l1-antibody-28-8-ab209959.html
The manufacturer has validated this antibody for FC in the species human and mouse.
5) anti-PD-L1 (Alexa Fluor 647) (abcam, ab209960, Rb) [28-8]:
https://www.abcam.cn/alexa-fluor-647-pd-l1-antibody-28-8-extracellular-domain-ab209960.html
The manufacturer has validated this antibody for FC in the species human and mouse.
6) Isotype Control (Alexa Fluor 488 Rabbit IgG) (abcam, ab199091, Rb) [EPR25A]:
https://www.abcam.cn/alexa-fluor-488-rabbit-igg-monoclonal-epr25a-isotype-control-ab199091.html
The manufacturer has validated this antibody for FC in the species human and mouse.
7) Isotype Control (Alexa Fluor 647 Rabbit IgG) (abcam, ab199093, Rb) [EPR25A]:
https://www.abcam.cn/alexa-fluor-647-rabbit-igg-monoclonal-epr25a-isotype-control-ab199093.html
The manufacturer has validated this antibody for FC in the species human and mouse.
8) goat anti-rabbit IgG H&L(Alexa Fluor 488) (abcam, ab150077):
https://www.abcam.cn/goat-rabbit-igg-hl-alexa-fluor-488-ab150077.html
The manufacturer has validated this antibody for IF in the species human.
9) goat anti-rabbit IgG H&L(Alexa Fluor 594) (abcam, ab150080):
https://www.abcam.cn/goat-rabbit-igg-hl-alexa-fluor-594-ab150080.html
The manufacturer has validated this antibody for IF in the species human.
10) goat anti-mouse IgG H&L(Alexa Fluor 488) (abcam, ab150113):
https://www.abcam.cn/goat-mouse-igg-hl-alexa-fluor-488-ab150113.html
The manufacturer has validated this antibody for IF in the species human.
11) goat anti-mouse IgG H&L(Alexa Fluor 594) (abcam, ab150116):
https://www.abcam.cn/goat-mouse-igg-hl-alexa-fluor-594-ab150116.html
The manufacturer has validated this antibody for IF in the species human.
12) anti-LAMP1 (Cell Signaling Technology, 9091, Rb) [D2D11]:
https://www.cellsignal.com/products/primary-antibodies/lamp1-d2d11-xp-rabbit-mab/9091
The manufacturer has validated this antibody for IF and WB in the species human.
13) anti-Lamin B1 (Cell Signaling Technology, 12586, Rb) [D4Q4Z]:
https://www.cellsignal.com/products/primary-antibodies/lamin-b1-d4q4z-rabbit-mab/12586
The manufacturer has validated this antibody for WB in the species human.
14) anti-Vinculin (Cell Signaling Technology, 13901, Rb) [E1E9V]:
https://www.cellsignal.com/products/primary-antibodies/vinculin-e1e9v-xp-rabbit-mab/13901
The manufacturer has validated this antibody for WB in the species human.
15) anti-ubiquitin (Cell Signaling Technology, 58395, Rb):
https://www.cellsignal.com/products/primary-antibodies/ubiquitin-p37-antibody/58395
The manufacturer has validated this antibody for WB in the species human.
16) anti-TOM20 (Cell Signaling Technology, 42406, Rb) [D8T4N]:
https://www.cellsignal.com/products/primary-antibodies/tom20-d8t4n-rabbit-mab/42406
The manufacturer has validated this antibody for WB in the species human.
17) anti-Calnexin (Cell Signaling Technology, 2679, Rb) [C5C9]:
https://www.cellsignal.com/products/primary-antibodies/calnexin-c5c9-rabbit-mab/2679
The manufacturer has validated this antibody for WB in the species human.
18) anti-αTubulin (Cell Signaling Technology, 2125, Rb) [11H10]:
https://www.cellsignal.com/products/primary-antibodies/a-tubulin-11h10-rabbit-mab/2125
The manufacturer has validated this antibody for WB in the species human.
19) anti-His-Tag (Abmart, M20001, Ms) [2A8]:
http://www.ab-mart.com.cn/page.aspx?node=%2060%20&id=%20959
The manufacturer has validated this antibody for WB in the species human.
20) anti-Myc-Tag (Abmart, M20002, Ms) [19C2]:
http://www.ab-mart.com.cn/page.aspx?node=%2060%20&id=%20962
The manufacturer has validated this antibody for WB in the species human.
21) anti-HA-Tag (Abmart, M20003, Ms) [26D11]:
http://www.ab-mart.com.cn/page.aspx?node=%2060%20&id=%20963
The manufacturer has validated this antibody for WB in the species human.
22) anti-DYKDDDDK-Tag (Abmart, M20008, Ms) [3B9]
http://www.ab-mart.com.cn/page.aspx?node=%2060%20&id=%20968
The manufacturer has validated this antibody for WB in the species human.
23) anti-GAPDH (Abmart, M20050, Ms) [3F10]
http://www.ab-mart.com.cn/page.aspx?node=%2059%20&id=%201247

The manufacturer has validated this antibody for WB in the species human.
25) HRP goat anti-mouse IgG (Bioker, BK-M050) ：
http://bioker.hzjxsj.com/home/product/detail.html?id=131109
The manufacturer has validated this antibody for WB.
26) HRP goat anti-rabbit IgG (Bioker, BK-R050):
http://bioker.hzjxsj.com/home/product/detail.html?id=131108
The manufacturer has validated this antibody for WB.
27) anti-PD-L1 (Proteintech, 17952-1-AP, Rb):
https://www.ptgcn.com/products/CD274-Antibody-17952-1-AP.htm
The manufacturer has validated this antibody for IP and WB in species human and mouse.
28) anti-PD-L1 (Proteintech, 66248-1-Ig, Ms) [2B11D11]:
https://www.ptgcn.com/products/PD-L1-CD274-Antibody-66248-1-Ig.htm
The manufacturer has validated this antibody for WB and IF in species human and mouse.
29) anti-CD8-APC (Proteintech, APC-65069, Rat) [53-6.7]:
https://www.ptgcn.com/products/CD8--Antibody-APC-65069.htm
The manufacturer has validated this antibody for FC in species human and mouse.
30) anti-CD3-APC-A750 (eBioscience, 47-0032, Rat) [17A2]:
https://www.thermofisher.cn/cn/zh/antibody/product/CD3-Antibody-clone-17A2-Monoclonal/47-0032-80
The manufacturer has validated this antibody for FC in species human and mouse.
31) anti-GzmB-FITC (eBioscience, 11-8898, Rat) [NGZB]:
https://www.thermofisher.cn/cn/zh/antibody/product/Granzyme-B-Antibody-clone-NGZB-Monoclonal/11-8898-80
The manufacturer has validated this antibody for FC in species human and mouse.
32) anti-CD3 (eBioscience, 16-0037, Ms) [OKT3]
https://www.thermofisher.cn/cn/zh/antibody/product/CD3-Antibody-clone-OKT3-Monoclonal/16-0037-85
This antibody for activation of PBMC was described in PMID: 30118680.
33) anti-CD4-PE (eBioscience, 12-0041, Rat) [GK1.5]:
https://www.thermofisher.cn/cn/zh/antibody/product/CD4-Antibody-clone-GK1-5-Monoclonal/12-0041-82
The manufacturer has validated this antibody for FC in species human and mouse.
34) anti-Fxop3-APC (eBioscience, 17-5773, Rat) [FJK-16s]:
https://www.thermofisher.cn/cn/zh/antibody/product/FOXP3-Antibody-clone-FJK-16s-Monoclonal/17-5773-82
The manufacturer has validated this antibody for FC in species human and mouse.
35) anti-CD11b-PE (eBioscience, 12-0112, Rat) [M1/70]:
https://www.thermofisher.cn/cn/zh/antibody/product/CD11b-Antibody-clone-M1-70-Monoclonal/12-0112-82
The manufacturer has validated this antibody for FC in species human and mouse.
36) anti-CD45-PB450 (Biolegend, 103126, Rat) [30-F11]:
https://www.biolegend.com/en-us/products/pacific-blue-anti-mouse-cd45-antibody-3102
The manufacturer has validated this antibody for FC in species human and mouse.
37) anti-CD3-APC (Biolegend, 100235, Rat) [17A2]:
https://www.biolegend.com/en-us/products/apc-anti-mouse-cd3-antibody-8055
The manufacturer has validated this antibody for FC in species human and mouse.
38) anti-CD8α-APC (Biolegend, 300912, Rat) [HIT8a]:
https://www.biolegend.com/en-us/products/apc-anti-human-cd8a-antibody-759
The manufacturer has validated this antibody for FC in species human and mouse.
39) anti-TIM3-PE (Biolegend, 345006, Rat) [F38-2E2]:
https://www.biolegend.com/en-us/products/pe-anti-human-cd366-tim-3-antibody-6121
The manufacturer has validated this antibody for FC in species human and mouse.
40) anti-NK1.1-PE (Biolegend, 108707, Rat) [PK136]:
https://www.biolegend.com/en-us/products/pe-anti-mouse-nk-1-1-antibody-431
The manufacturer has validated this antibody for FC in species human and mouse.
41) anti-F/480-APC (Biolegend, 123116, Rat) [BM8]:
https://www.biolegend.com/en-us/products/apc-anti-mouse-f4-80-antibody-4071
The manufacturer has validated this antibody for FC in species human and mouse.
42) anti-Gr1-APC (Biolegend, 108424, Rat) [RB6-8C5]:
https://www.biolegend.com/en-us/products/apc-cyanine7-anti-mouse-ly-6gly-6c-gr-1-antibody-3935
The manufacturer has validated this antibody for FC in species human and mouse.
43) InVivoMAb anti-mouse CTLA-4 (Bio X Cell, BE0032) [UC10-4F10-11]:
https://bxcell.com/product/m-cd152-m-ctla-4-2/
The manufacturer has validated this antibody for in vivo use in species mouse.
44) InVivoMAb anti-mouse NK1.1 (Bio X Cell, BE0036) [PK136]
https://bxcell.com/product/nk-1-1/
The manufacturer has validated this antibody for in vivo use in species mouse.
45) InVivoMAb anti-mouse CD8α (Bio X Cell, BE0117) [YTS 169.4]:
https://bxcell.com/product/m-cd8/
The manufacturer has validated this antibody for in vivo use in species mouse.
46) InVivoMAb anti-mouse CD4 (Bio X Cell, BE0003-1) [GK1.5]:
https://bxcell.com/product/m-cd4/
The manufacturer has validated this antibody for in vivo use in species mouse.
47) InVivoMAb anti-mouse CD4 (Bio X Cell, BE0003-1) [GK1.5]:

https://www.scbt.com/zh/p/itm1-antibody-a-2?requestFrom=search
The manufacturer has validated this antibody for WB in species human.
48) anti-CMTM4 (Huabio, ER63053, Rb):
http://www.huabio.cn/product/CMTM4-antibody-ER63053
The manufacturer has validated this antibody for WB in species human.
49) anti-CMTM6 (Huabio, ER65542, Rb).
http://www.huabio.cn/product/CMTM6-antibody-ER65542
The manufacturer has validated this antibody for WB in species human.

Eukaryotic cell lines

Policy information about cell lines and Sex and Gender in Research

Cell line source(s)	Human breast cancer cell lines MDA-MB-231 (CRM-HTB-26; RRID: CVCL_0062), MDA-MB-468 (HTB-132; RRID: CVCL_0419), the human embryonic kidney cell line HEK293T (CRL-3216; RRID: CVCL_0063), the human lung cancer cell line A549 (CCL-185; RRID: CVCL_0023), the human liver cancer cell line HepG2 (HB-8065; RRID: CVCL_0027), the human gastric cancer cell line AGS (CRL-1739; RRID: CVCL_0139), the mouse breast cancer cell line 4T1 (CRL-2539; RRID: CVCL_0125) and the mouse breast cancer cell line EO771 (CRL-3461; RRID: CVCL_GR23) were purchased from the American type culture collection (ATCC). The mouse gastric cancer cell line MFC (1101MOU-PUMC000143; RRID: CVCL_5J48) was purchased from Chinese National Infrastructure of Cell Line Resource (Beijing, China).
Authentication	All cell lines were authenticated based on STR fingerprinting before use.
Mycoplasma contamination	All cell lines were tested negative for mycoplasma contamination. Stated in Methods section.
Commonly misidentified lines (See ICLAC register)	No commonly misidentified cell lines were used.

Animals and other research organisms

Policy information about studies involving animals; ARRIVE guidelines recommended for reporting animal research, and Sex and Gender in Research

Laboratory animals	Female mice (Balb/c, Balb/c nude or C57BL/6J strain; 4–5 weeks old) were purchased from the Shanghai Laboratory Animals Center and used in the xenograft mouse model assay. Animals were housed in a pathogen-free barrier environment (around 20 °C with 40% humidity and 12-h dark/light cycle) throughout the study. Mice were fed a normal chow diet and water with ad libitum feeding. Control and experimental animals were bred separately.
Wild animals	This study did not involve the wild animals.
Reporting on sex	Since this study focused on breast cancer models, only female mice were used as study subjects.
Field-collected samples	This study did not include field-collected samples.
Ethics oversight	Animals were housed in a pathogen-free barrier environment (around 20 °C with 40% humidity and 12-h dark/light cycle) throughout the study, and experimental protocols were approved by the Animal Care and Use Committee of Zhejiang University School of Medicine (ZJU20210045).

Note that full information on the approval of the study protocol must also be provided in the manuscript.

Flow Cytometry

Plots

Confirm that:

- [x] The axis labels state the marker and fluorochrome used (e.g. CD4-FITC).
- [x] The axis scales are clearly visible. Include numbers along axes only for bottom left plot of group (a 'group' is an analysis of identical markers).
- [x] All plots are contour plots with outliers or pseudocolor plots.
- [x] A numerical value for number of cells or percentage (with statistics) is provided.

Methodology

Sample preparation	Tumor tissues were cut into pieces and suspended with 5 ml tumor digestion buffer (5% FBS, 20 mM glutamine, 50 μM β-mercaptoethanol, 1.6 mg ml−1 collagenase IV, 1.6 mg ml−1 collagenase I and 0.02% DNase I). After rotation for 1.5 h at 37°C, tissues were digested. The cell suspension was filtered using a 70-μm filter to obtain a single-cell suspension. Leukocytes were isolated by density-gradient centrifugation using 40% and 70% Percoll (GE). Then, tumor-infiltrating leukocytes were

	stained using fluorescently labeled antibodies for different markers, such as CD8, CD3, GzmB.
Instrument	CytoFlex analyzer (Beckman Coulter)
Software	FlowJo X, CytExpert v2.3 and GraphPad Prism 8.0
Cell population abundance	Moderate
Gating strategy	In our experiment, zombie was used to gating the living cells, APC-A750 conjugated-CD3 antibody was used to gate the T cells, APC conjugated-CD8 antibody was used to circle the Tc cells, and FITC conjugated-GzmB antibody was used to gate the activated Tc cells. PB-450 conjugeated-CD45 antibody was used to gate the lymphocyte, PE conjugated-NK 1.1 antibody and APC conjugated-CD3 antibody was used to circle the NK cells, PE conjugated-CD4 antibody and APC conjugated-CD3 antibody was used to circle the CD4 T cells, PE conjugated-CD4 antibody and APC conjugated-Foxp3 antibody was used to circle the Treg cells, PE conjugated-TIM3 and APC conjugated-CD8 antibody was used to circle exhausted T cell, PE conjugated-CD11b antibody and APC-conjugated F/480 antibody was used to circle the TAM cells, and PE conjugated-CD11b antibody and APC-conjugated Gr1 antibody was used to circle the MDSC.

☒ Tick this box to confirm that a figure exemplifying the gating strategy is provided in the Supplementary Information.
